# A fully probabilistic control framework for stochastic systems with input and state delay

**DOI:** 10.1038/s41598-022-11514-z

**Published:** 2022-05-12

**Authors:** Randa Herzallah, Yuyang Zhou

**Affiliations:** Mathematics Group, Aston University, Aston Triangle, Birmingham, B4 7ET UK

**Keywords:** Engineering, Mathematics and computing

## Abstract

This paper proposes a unified probabilistic control framework for a class of stochastic systems with both control input and state time delays. Both of the stochastic nature and time delays in the system dynamics are considered simultaneously, thus providing a comprehensive and rigorous control methodology. The problem is formulated in a fully probabilistic framework, where the system dynamics and its controller are fully characterised by arbitrary probabilistic models. In this framework, the Kullback–Leibler Divergence between the actual joint probability density function of the system dynamics and controller and a predefined ideal joint probability density function is used to characterise the discrepancy between the two distributions and derive the randomised controller. Time delays in the control input and system state are taken into consideration in the optimisation process for the derivation of the optimal randomised controller. Besides, the analytic control solution of the time delay fully probabilistic control problem for a class of linear Gaussian stochastic systems is derived while the successive approximation approach is implemented to deal with the time-advanced components in the control law that result from the existence of time delays. The effectiveness of the proposed control framework is then illustrated on a numerical example and a real-world example.

## Introduction

Time delay systems are ubiquitous in nature, appearing in a broad range of fields including engineering, mathematics, biology, ecology, and physics^1^. Time delays can be either inherent due to delays in the system components or originate from propagation phenomena, material or energy transfer in interconnected systems, data transmission in communication systems, or the implementation of feedback loop. While delays might have a stabilising effect, they are also normally the main sources inducing oscillations, instability and degrading the system performance^2,3^. From the control point of view, they make the controller design and system analysis more complicated. The first effective control scheme for stable time-delay systems is the Smith predictor^4^. It was then extended to control unstable time-delay systems^5^. Since then, the robust control of time-delay systems has attracted a lot of attention. This includes the stabilization and robust control of time-delay systems using Lyapunov theory and linear matrix inequality (LMI) tools. Quadratic stablisability and ${H_{\infty}}$ control has also been extensively used for controlling general time-delay systems^6–8^.

Furthermore, the recent emergence of new technologies and systems such as communication and information technologies, network-controlled systems, parallel computation, and multiagent systems emphasises the inevitable requirement for the characterisation and consideration of time delays. This has been highlighted in the tracking control problem for cloud robotic systems with delayed measurements^9^ and for droop controlled AC microgrid^10^. Similarly, traffic management systems to mitigate congestion in urban networks requires feedback gating control that considers time delays in the traffic system^11^. For multiagent systems such as drone formations for agriculture or surveillance, time-delay, random packet loss, and uncertainty of system model were identified to be among the most important challenges for the controller design^12^.

The demand for stable behaviour of time delay systems motivates the core of this article: to devise a theoretical and algorithmic framework for a probabilistic control approach that can consider the effect of time delays in real world dynamical systems and improve the performance of their behaviour.

However, time delays lead to many challenges, not least of which is how a controller can be designed without overreacting to overestimated errors. The challenge is to apply appropriate control actions that address long time delays, thus providing acceptable performance under these conditions. In addition to dealing with nonlinear behaviour of the system dynamics we have to contend with noise, uncertainty, and stochasticity across the system, so conventional deterministic control techniques cannot be employed to direct the behaviour of the time delay systems. This implies we need an approach where the system evolution and external control strategies need to be developed probabilistically.

Considering the limitations of the existing control methods, the focus of this article is to introduce the theoretical structure for a universal control intervention strategy that can effectively handle the stochastic nature of the controlled system and any delays affecting its dynamics. The novelty is in treating the full control problem as intrinsically probabilistic, where a probabilistic model is used to characterise the time evolution of the system dynamics and a probabilistic controller is devised.

## Review of current approaches

Time delays, which normally appear as transportation and communication lags and also arise as feedback delay in measurement and closed-loop systems, are commonly encountered in real-life practical systems including engineering, chemistry, biology, climatology, and economical systems^13–16^. Controlling and understanding time-delay systems have always been very challenging. For these systems, it is important that time delays are incorporated in the systems models and that they are considered in the theoritical and control analysis of the closed loop system behaviour^17^. Thus, a large and growing body of research on time delays and their compensation methods has been investigated and published. For instance, a graphical methodology to calculate the stabilizing values of PI controller parameters for a single-area load frequency control (LFC) system with time delay was proposed in^18^. In^16^, a sliding mode control design for fractional-order systems with input and state time-delay is presented. A delay discretization approach is introduced in^19^ to improve the tolerable state delay margin of the interconnected power system while^20^ investigated the event-triggered drive-response synchronization control for Takagi-Sugeno fuzzy neural networked systems with time delay. Some current results related to the control problem of time-delay systems have been summarised in^13,21,22^.

Despite all these significant efforts that have been investigated, most of the existing research has only considered either input time delay or state time delay in their models but not both^17,20,23^, thus limiting their implementation to real life situations. Moreover, a rich group of literature tried to solve the time delay issue by transforming discrete-time systems with time-delays into delay free systems by properly defining new state variables^23,37^. This approach however significantly increases the dimensionality of the controlled system, thus complicates the analysis of systems with long time delays and makes large-scale and high dimensional systems numerically demanding. Recent work has also addressed non-constant time delays where the time delays are considered to vary stochastically^24–27^. Most of the aforementioned approaches on the other hand were presented under the assumption that the dynamics of the systems being considered follow a deterministic description. Such an assumption however is not realistic since real-world processes are usually subjected to various sources of uncertainties including random noises, functional uncertainties, and disturbances introduced by measurement devices and other surrounding environmental conditions. For such systems which involve both high levels of uncertainties and input and/or state delays, the controller design becomes much more challenging.

To address the stochasticity of the systems dynamics, a new method which is based on the use of the Kullback Leibler divergence between probability density functions is proposed in^28,29^. This method is referred to as the fully probabilistic design (FPD) method. The conventional FPD method has then been applied and extended to various classes of stochastic systems in recent decades. For instance, in^30^, the conventional FPD has been modified and extended for a class of stochastic dynamic systems with multiplicative noises while^31^ has combined FPD method with disturbance observer based controller. For systems with delays, the work in^32^ extended the conventional FPD and proposed a Time Delay Fully Probabilistic Design (TDFPD) method for a class of stochastic systems with input delays. However, the TDFPD method proposed in^32^ only considered a single input delay in the system dynamics, which is very limiting for real-world applications.

As such, the objective of the current paper is to extend the method in^32^ by developing a probabilistic control framework for a class of stochastic systems that has multiple input and state time delays. Considering the stochastic nature of the class of systems under study, the proposed methodology characterises the dynamics of the system using probabilistic models. The framework then adopt and extend the probabilistic design method^32^ such that multiple state and input time delays are taken into consideration in the derivation of the optimal controller. Following this approach the optimal controller will be a randomised controller that minimizes the Kullback–Leibler divergence between the joint probability density function (pdf) of the system dynamics and a predefined desired joint pdf. As will be seen from further development, the derived fully probabilistic control solution in this paper (which will be referred to as the Multiple Time Delay Fully Probabilistic Control (MTDFPC)) has several advantages including the attainment of a closed form randomised optimal controllers, the consideration of systems noises and uncertainties in the system of dynamics, and the consideration of systems multiple time delays; all in a unified probabilistic framework. Due to the existence of the input and state delays, the obtained randomised optimal solutions contain both time-delay and time-advance terms, which is difficult to be solved analytically. To address this problem, we adopt the successive approximation approach (SAA)^33^ to solve the control problem. The advantage of this method is that it is suitable not only for small-time delays but also for large-time delays.

To re-emphasise, compared with the existing results on this topic, the contribution of the proposed framework in this paper can be summarised as follows. Firstly, considering the stochastic nature of the systems dynamics, a fully probabilistic control framework is developed which considers the uncertainties and noises in the system dynamics as well as the multiple time delays in the control input and system state. Unlike most of the existing literature where the system dynamics are described by deterministic equations, in our framework, the system dynamics are completely characteried by pdfs. Secondly, this framework takes both multiple input delays and multiple state delays into consideration, extending the TDFPD^32^ that only considers one single type of delay. The consideration of both input and state delays in the system models offers a more general and precise description of the real-world system dynamics. This is considered as the main contribution of this paper as only few existing proposed control algorithms considered both multiple input delays and multiple state delays. Thirdly, a numerical optimal solution can be obtained using the SAA, which provides an explicit control procedure to follow and to implement. Moreover, the SAA is also capable of dealing with systems containing long time delays, thus lifting the limitations of some existing methods.

## MTDFPC for stochastic systems with input and state delays

This section will formulate the control objective, provide the probabilistic description of the considered class of stochastic systems with multiple state and control input delays, and derive the general solution of the MTDFPC for this class of systems based on their arbitrary probabilistic description.

### Control objectives of the MTDFPC problem

In the fully probabilistic control design method^28^ the aim is to design a randomised controller that shapes the joint pdf describing the closed loop behaviour of the controlled system and makes it as close as possible to a predefined ideal joint pdf. In this method, the discrepancy between the two joint pdfs is measured by the Kullback Leibler divergence. The FPD method however insists on zero delay between the input and the system state, thus, does not provide optimal solutions for systems with delays. As such, this method will be extended in this section to consider stochastic systems with multiple control input and state delays.

Consider the following probabilistic description for the considered class of stochastic systems with multiple state and control input delays that can be represented at each time instant, *t* by the following conditional pdf,

1 $$\begin{aligned} s(x_{t}|x_{t-1}, u_t,x_{t-{h_1}}, \dots , x_{t-{h_{N_1}}}, u_{t-{L_1}}, \dots , u_{t-{L_{N_2}}}), \end{aligned}$$

where ${x_t} \in {R^n}$ represents the system state, ${u_t} \in {R^m}$ represents the control input, and $h_i, i=\{1, 2, \dots , N_1\}$ and $L_j, j=\{1, 2, \dots , N_2\}$ denote time delays in the state and input respectively. Also, assume that, $h=\underbrace{\text {max}}_i \{h_i\}$ and $L=\underbrace{\text {max}}_j \{L_j\}$, then the initial values $x_i, i=-h,\dots , 0$, $u_j, j= -L, \dots , 0$, are known.

For the formulation in this paper *s*(.|.) does not need to be known and does not need to be constrained by the Gaussian assumption. Also, note that because of the stochastic nature of the considered class of systems with time delays, the probabilistic description of the system dynamics as given in (1) provides a complete specification of the present state conditioned on the previous state and present and previous control. To reemphasise, the probabilistic description (1) is general and can be characterised from the underlying stochastic evolution of the system dynamics. The formulation in this section will be based on this general probabilistic description. The results obtained here will then be demonstrated in the following sections on a class of stochastic linear time delay systems with additive Gaussian noise. However, this formulation is not restricted by the assumption of the additive noise nor it is restricted by the linearity of the system. The noise could be multiplicative and the system equation could be nonlinear.

For these stochastic systems, the closed loop behaviour of the system dynamics can be specified by the joint probability density function of the system state and control input. This joint pdf of the closed loop dynamics of the system provides the most complete description of its behaviour. As such, similar to the conventional FPD approach, the objective of the MTDFPC control problem is specified as the design of a randomised controller, $c(u_t|x_{t-1}, x_{t-{h_1}}, \dots , x_{t-{h_{N_1}}}, u_{t-{L_1}}, \dots , u_{t-{L_{N_2}}})$ that minimises the Kullback–Leibler divergence between the joint pdf of the closed loop description of the system dynamics, $f(\mathscr{X}(t,T))$ and a predefined ideal joint pdf $\,^{{I}}f(\mathscr{X}(t,T))$,

2 $$\begin{aligned} \mathscr{D}\left( f||\,^{{I}}f\right) \equiv \int f(\mathscr{X}(t,T))\ln \left( \frac{f(\mathscr{X}(t,T))}{\,^{{I}}f(\mathscr{X}(t,T))}\right) \,\mathrm {d} \mathscr{X}(t,T), \end{aligned}$$

where $\mathscr{X}(t,T) = \{x_{t}, \dots , x_T,u_{t}, \dots , u_T\}$ is the closed loop observed data sequence, and $T \le \infty$ is a given control horizon. For stochastic systems with multiple input and state delays given in (1), the joint pdf of the system dynamics, $f(\mathscr{X}(t,T))$ can be evaluated using the chain rule^34^ as follows,

3 $$\begin{aligned} f(\mathscr{X}(t,T)) =&\prod _{t=1}^T s(x_{t}|x_{t-1}, u_t,x_{t-{h_1}}, \dots , x_{t-{h_{N_1}}}, u_{t-{L_1}}, \dots , u_{t-{L_{N_2}}}) \\ \nonumber& c(u_t|x_{t-1}, x_{t-{h_1}}, \dots , x_{t-{h_{N_1}}}, u_{t-{L_1}}, \dots , u_{t-{L_{N_2}}}), \end{aligned}$$

where the pdf $s(x_{t}|x_{t-1}, u_t,x_{t-{h_1}}, \dots , x_{t-{h_{N_1}}}, u_{t-{L_1}}, \dots , u_{t-{L_{N_2}}})$ describes the dynamics of the observed state vector $x_{t}$, and $c(u_t|x_{t-1}, x_{t-{h_1}}, \dots , x_{t-{h_{N_1}}}, u_{t-{L_1}}, \dots , u_{t-{L_{N_2}}})$ represents the pdf of the required randomised controller as mentioned earlier.

Similarly, the ideal joint pdf of the closed loop data can be factorised as follows,

4 $$\begin{aligned} \,^{{I}}f(\mathscr{X}(t,T)) =&\prod _{t=1}^T \,^{{I}}s(x_{t}|x_{t-1}, u_t,x_{t-{h_1}}, \dots , x_{t-{h_{N_1}}}, u_{t-{L_1}}, \dots , u_{t-{L_{N_2}}})\,^{{I}} \\ \nonumber& c(u_t|x_{t-1}, x_{t-{h_1}}, \dots , x_{t-{h_{N_1}}}, u_{t-{L_1}}, \dots , u_{t-{L_{N_2}}}), \end{aligned}$$

where the pdf $\,^{{I}}s(x_{t}|x_{t-1}, u_t,x_{t-{h_1}}, \dots , x_{t-{h_{N_1}}}, u_{t-{L_1}}, \dots , u_{t-{L_{N_2}}})$ describes the ideal distribution of the system state vector $x_{t}$, and $\,^{{I}}c(u_t|x_{t-1}, x_{t-{h_1}}, \dots , x_{t-{h_{N_1}}}, u_{t-{L_1}}, \dots , u_{t-{L_{N_2}}})$ represents the ideal pdf of the randomised controller. Given the definitions of the joint pdf of the closed loop system and the ideal pdf as specified by Eqs.(3) and (4) respectively, minimisation of (2) can be obtained recursively by introducing the following definition,

5 $$\begin{aligned}{}&-\ln (\gamma (x_{t-1}))= \min _{c(u_t|x_{t-1}, x_{t-{h_1}}, \dots , x_{t-{h_{N_1}}}, u_{t-{L_1}}, \dots , u_{t-{L_{N_2}}})} \sum _{\tau =t}^T \int f(\mathscr{X}_\tau ,\dots ,\mathscr{X}_T|x_{t-1})\nonumber \\&\times \bigg [ \ln \left( \frac{s(x_{\tau }|x_{\tau -1}, u_\tau ,x_{\tau -{h_1}}, \dots , x_{\tau -{h_{N_1}}}, u_{\tau -{L_1}}, \dots , u_{\tau -{L_{N_2}}})}{\,^{{I}}s(x_{\tau }|x_{\tau -1}, u_\tau ,x_{\tau -{h_1}}, \dots , x_{\tau -{h_{N_1}}}, u_{\tau -{L_1}}, \dots , u_{\tau -{L_{N_2}}})} \right) \nonumber \\&+ \ln \left( \frac{c(u_\tau |x_{\tau -1}, x_{\tau -{h_1}}, \dots , x_{\tau -{h_{N_1}}}, u_{\tau -{L_1}}, \dots , u_{\tau -{L_{N_2}}})}{\,^{{I}}c(u_\tau |x_{\tau -1}, x_{\tau -{h_1}}, \dots , x_{\tau -{h_{N_1}}}, u_{\tau -{L_1}}, \dots , u_{\tau -{L_{N_2}}})} \right) \bigg ]\mathrm {d}(\mathscr{X}_\tau ,\dots ,\mathscr{X}_T), \end{aligned}$$

for arbitrary $\tau \in \{1,\dots ,T\}$. Here $\mathscr{X}_t= (x_t,u_t)$, and $-\ln (\gamma (x_{t-1}))$ is the optimal performance index.

The definition in Eq. (5) leads to the recursive formula for the cost function specified in the following theorem. This recursive formula will be used later for the derivation of the optimal randomised controller.

#### Theorem 1

Using the definition given in 5 the minimisation of the Kullback–Leibler divergence 2 can be performed recursively to give the following recurrence functional equation,

6 $$\begin{aligned}{}&-\ln (\gamma (x_{t-1}))= \min _{c(u_t|x_{t-1}, x_{t-{h_1}}, \dots , x_{t-{h_{N_1}}}, u_{t-{L_1}}, \dots , u_{t-{L_{N_2}}})} \int s(x_{t}|x_{t-1}, u_t,x_{t-{h_1}}, \dots , x_{t-{h_{N_1}}}, u_{t-{L_1}}, \dots , u_{t-{L_{N_2}}}) \nonumber \\&\times c(u_t|x_{t-1}, x_{t-{h_1}}, \dots , x_{t-{h_{N_1}}}, u_{t-{L_1}}, \dots , u_{t-{L_{N_2}}}) \nonumber \\&\bigg [ \underbrace{\ln \left( \frac{s(x_{t}|x_{t-1}, u_t,x_{t-{h_1}}, \dots , x_{t-{h_{N_1}}}, u_{t-{L_1}}, \dots , u_{t-{L_{N_2}}})c(u_t|x_{t-1}, x_{t-{h_1}}, \dots , x_{t-{h_{N_1}}}, u_{t-{L_1}}, \dots , u_{t-{L_{N_2}}})}{\,^{{I}}s(x_{t}|x_{t-1}, u_t,x_{t-{h_1}}, \dots , x_{t-{h_{N_1}}}, u_{t-{L_1}}, \dots , u_{t-{L_{N_2}}})\,^{{I}}c(u_t|x_{t-1}, x_{t-{h_1}}, \dots , x_{t-{h_{N_1}}}, u_{t-{L_1}}, \dots , u_{t-{L_{N_2}}})} \right) }_{\equiv { partial cost}\ \Longrightarrow U(x_{t},u_{t})} \nonumber \\&-\ln (\gamma (x_{t})) - \sum _{i=1}^{N_1} \int s(x_{t+h_i}|x_{t+h_i-1}, u_{t+h_i},x_{t+h_i-{h_1}}, \dots , x_{t+h_i-{h_{N_1}}}, u_{t+h_i-{L_1}}, \dots , u_{t+h_i-{L_{N_2}}}) \nonumber \\&\times c(u_{t+h_i}|x_{t+h_i-1}, x_{t+h_i-{h_1}}, \dots , x_{t+h_i-{h_{N_1}}}, u_{t+h_i-{L_1}}, \dots , u_{t+h_i-{L_{N_2}}}) \ln (\gamma (x_{t+h_i})) \mathrm {d}(x_{t+h_i}, u_{t+h_i}) \delta (t+h_i) \nonumber \\&- \sum _{j=1}^{N_2} \int s(x_{t+L_j}|x_{t+L_j-1}, u_{t+L_j},x_{t+L_j-{h_1}}, \dots , x_{t+L_j-{h_{N_1}}}, u_{t+L_j-{L_1}}, \dots , u_{t+L_j-{L_{N_2}}}) \nonumber \\&\times c(u_{t+L_j}|x_{t+L_j-1}, x_{t+L_j-{h_1}}, \dots , x_{t+L_j-{h_{N_1}}}, u_{t+L_j-{L_1}}, \dots , u_{t+L_j-{L_{N_2}}}) \ln (\gamma (x_{t+L_j})) \mathrm {d}(x_{t+L_j}, u_{t+L_j}) \delta (t+L_j)\bigg ] \nonumber \\&\,\mathrm {d}(x_{t},u_{t}), \end{aligned}$$

where,

7 $$\begin{aligned} \delta (t) = \left\{ \begin{array}{ll} 0, &{}\quad t = T, T+1, \dots \\ 1, &{}\quad t = 0, 1, 2, T-1.\end{array} \right. \end{aligned}$$

#### Proof

The proof is given in the Supplementary Appendix. $\square$

### Solution to the MTDFPC for arbitrary density functions

The general solution of the optimised MTDFPC control problem as obtained from the minimisation of the cost-to-go function defined in Eq. (6) with respect to randomised control input, $c(u_t|x_{t-1}, x_{t-{h_1}}, \dots , x_{t-{h_{N_1}}}, u_{t-{L_1}}, \dots , u_{t-{L_{N_2}}})$ can be shown to be given by the following theorem.

#### Theorem 2

The pdf of the optimal randomised controller minimising the cost–to–go function (6) subject to the conditional distribution of the stochastic system, $s(x_{t}|x_{t-1}, u_t,x_{t-{h_1}}, \dots , x_{t-{h_{N_1}}}, u_{t-{L_1}}, \dots , u_{t-{L_{N_2}}})$ is given by,

8 $$\begin{aligned}{}&c(u_t|x_{t-1}, x_{t-{h_1}}, \dots , x_{t-{h_{N_1}}}, u_{t-{L_1}}, \dots , u_{t-{L_{N_2}}}) \nonumber \\&= \frac{\,^{{I}}c(u_t|x_{t-1}, x_{t-{h_1}}, \dots , x_{t-{h_{N_1}}}, u_{t-{L_1}}, \dots , u_{t-{L_{N_2}}}) \exp \bigg [-\beta _1(.)-\beta _2(.)-\tilde{\beta }_3(.) -\tilde{\beta }_4(.) \bigg ]}{\gamma (x_{t-1})}, \end{aligned}$$

where,

9 $$\begin{aligned}{}&\gamma (x_{t-1}) = \int \,^{{I}}c(u_{t}|x_{t-1},u_{t-h}) \exp \bigg [-\beta _1(.)-\beta _2(.) - \tilde{\beta }_3(.) -\tilde{\beta }_4(.) \bigg ] \mathrm {d} u_{t}, \nonumber \\&\tilde{\beta }_3(.) = \int c(u_{t+h_i}|x_{t+h_i-1}, x_{t+h_i-{h_1}}, \dots , x_{t+h_i-{h_{N_1}}}, u_{t+h_i-{L_1}}, \dots , u_{t+h_i-{L_{N_2}}}) \beta _3(.) \delta (t+h_i) \mathrm {d} u_{t+h_i}, \nonumber \\&\tilde{\beta }_4(.) = \int c(u_{t+L_j}|x_{t+L_j-1}, x_{t+L_j-{h_1}}, \dots , x_{t+L_j-{h_{N_1}}}, u_{t+L_j-{L_1}}, \dots , u_{t+L_j-{L_{N_2}}}) \beta _4(.) \delta (t+L_j) \mathrm {d} u_{t+L_j}, \end{aligned}$$

10 $$\begin{aligned}{}&\beta _1(.) = \int s(x_{t}|x_{t-1}, u_t,x_{t-{h_1}}, \dots , x_{t-{h_{N_1}}}, u_{t-{L_1}}, \dots , u_{t-{L_{N_2}}}) \nonumber \\&\times \bigg [\ln \frac{s(x_{t}|x_{t-1}, u_t,x_{t-{h_1}}, \dots , x_{t-{h_{N_1}}}, u_{t-{L_1}}, \dots , u_{t-{L_{N_2}}})}{\,^{{I}}s(x_{t}|x_{t-1}, u_t,x_{t-{h_1}}, \dots , x_{t-{h_{N_1}}}, 
u_{t-{L_1}}, \dots , u_{t-{L_{N_2}}})} \bigg ] \mathrm {d} x_{t}, \nonumber \\&\beta _2(.) = -\int s(x_{t}|x_{t-1}, u_t,x_{t-{h_1}}, \dots , x_{t-{h_{N_1}}}, u_{t-{L_1}}, \dots , u_{t-{L_{N_2}}}) \ln (\gamma (x_{t})) \mathrm {d} x_{t}, \nonumber \\&\beta _3(.) = - \sum _{i=1}^{N_1} \int s(x_{t+h_i}|x_{t+h_i-1}, u_{t+h_i},x_{t+h_i-{h_1}}, \dots , x_{t+h_i-{h_{N_1}}}, u_{t+h_i-{L_1}}, \dots , u_{t+h_i-{L_{N_2}}}) \nonumber \\&\times s(x_{t}|x_{t-1}, u_t,x_{t-{h_1}}, \dots , x_{t-{h_{N_1}}}, u_{t-{L_1}}, \dots , u_{t-{L_{N_2}}}) \ln (\gamma (x_{t+h_i})) \mathrm {d} (x_t,x_{t+h_i}), \nonumber \\&\beta _4(.) = - \sum _{j=1}^{N_2} \int s(x_{t+L_j}|x_{t+L_j-1}, u_{t+L_j},x_{t+L_j-{h_1}}, \dots , x_{t+L_j-{h_{N_1}}}, u_{t+L_j-{L_1}}, \dots , u_{t+L_j-{L_{N_2}}}) \nonumber \\&\times s(x_{t}|x_{t-1}, u_t,x_{t-{h_1}}, \dots , x_{t-{h_{N_1}}}, u_{t-{L_1}}, \dots , u_{t-{L_{N_2}}}) \ln (\gamma (x_{t+L_j})) \mathrm {d} (x_t,x_{t+L_j}), \end{aligned}$$

for $t=0, 1, \dots , T$, and $\gamma (x_{T})=1$. Also note that the dot argument in the $\beta$’s can be deduced from their corresponding equations. It basically consists of all the variables the are not integrated over.

#### Proof

The proof of this theorem is given in the Supplementary Appendix. $\square$

## Solution to the MTDFPC for Gaussian probabilistic state space models

Theorem 2 provides the general solution for the considered class of stochastic systems with multiple input and state delays that can be described by arbitrary pdfs. This general solution however needs to be evaluated numerically if the systems distributions contain non-linearity and non-Gaussianity. Nonetheless, the Computation of the solution numerically yields high computational costs that increase with the complexity and dimensionality of the problem. Therefore, to facilitate the understanding and the derivation of an analytical solution for the proposed probabilistic control framework, the solution stated in Theorem 2 will be applied here to a class of linear and Gaussian, stochastic dynamical systems that are also affected by multiple input and state delays. This class of linear stochastic systems that are driven by multiple input and state delays is described by,

11 $$\begin{aligned} {x_t} = A{x_{t - 1}} + \sum \limits _{i = 1}^{{N_1}} {{A_i}{x_{t - {h_i}-1}}} + B{u_t} + \sum \limits _{j = 1}^{{N_2}} {{B_j}{u_{t - {L_j}}}} + \varepsilon _t, \end{aligned}$$

where *A* is the system state matrix, *B* is the control input matrix, $A_i$, $i=1,...,N_1$ represent the matrices of delayed system state, and $B_j$, $j=1,...,N_2$ denote the matrices of delayed input. In addition, $h_i, i=\{1, 2, \dots , N_1\}$ and $L_j, j=\{1, 2, \dots , N_2\}$ are time delays in the state and input as discussed before. Moreover, ${\varepsilon_t}$ is a zero mean Gaussian noise with covariance *Q*. As discussed earlier, the effect of the noise, ${\varepsilon _t}$ on the system state $x_t$ means that complete specification of the system state can be only achieved through its probability distribution conditioned on the current control input and previous state and control input. For the class of linear systems given in (11) the generative probabilistic model of the system state can be characterised by a Gaussian distribution as follows,

12 $$\begin{aligned} s(x_{t}|x_{t-1}, u_t,x_{t-{h_1}-1}, \dots , x_{t-{h_{N_1}}-1}, u_{t-{L_1}}, \dots , u_{t-{L_{N_2}}})\sim N(\mu _{t},{Q}), \end{aligned}$$

where $\mu _{t}=A{x_{t - 1}} + \sum \limits _{i = 1}^{{N_1}} {{A_i}{x_{t - {h_i}-1}}} + B{u_t} + \sum \limits _{j = 1}^{{N_2}} {{B_j}{u_{t - {L_j}}}}$ is the mean of the system state at time *t*.

As discussed in the previous section, the control objective within the MTDFPC framework can be achieved by specifying the appropriate parameters of the ideal distribution that reflects the desired objective. In this paper, a tracking problem where the controller is designed to make the state of the system given by (11) follows a predefined reference state is considered. Thus, for the probabilistic description of the system given in (12) the ideal distribution of the system state is taken to have the following form,

13 $$\begin{aligned} {}^Is(x_{t}|x_{t-1}, u_t,x_{t-{h_1}-1}, \dots , x_{t-{h_{N_1}-1}}, u_{t-{L_1}}, \dots , u_{t-{L_{N_2}}})&\sim N(x_{r},{R}) , \end{aligned}$$

where $x_r$ denotes the predefined reference state for the system state to track, and *R* is the ideal covariance determining the spread of the state values around the desired reference state.

Similarly, the ideal distribution of the controller is specified as follows,

14 $$\begin{aligned} {}^Ic(u_t|x_{t-1}, x_{t-{h_1}-1}, \dots , x_{t-{h_{N_1}-1}}, u_{t-{L_1}}, \dots , u_{t-{L_{N_2}}})&\sim N(u_{r},\Gamma ) , \end{aligned}$$

where $\Gamma$ is the covariance of the ideal distribution of the controller which indicates the admissible range of the optimal control input and $u_{r}$ is the mean of the ideal distribution of the randomised controller which can be calculated from the limit of the expected value of the state at time *t*, $E[x_t]$ as *t* approaches infinity,

$$\begin{aligned}{}&\mathop {\lim }\limits _{t \rightarrow \infty } E[{x_t}] = \mathop {\lim }\limits _{t \rightarrow \infty } (A{x_{t - 1}} + \sum \limits _{i = 1}^{{N_1}} {{A_i}{x_{t - {h_i} - 1}}} + B{u_t} + \sum \limits _{j = 1}^{{N_2}} {{B_j}}{{u_{t - {L_j}}}} ). \end{aligned}$$

By noting that $\mathop {\lim }\limits _{t \rightarrow \infty } {x_t} = \mathop {\lim }\limits _{t \rightarrow \infty } x_{t - {h_i} - 1} = x_r$, and $\mathop {\lim }\limits _{t \rightarrow \infty } {u_t} = \mathop {\lim }\limits _{t \rightarrow \infty } {u_{t - {L_j}}} = u_r$, one gets,

15 $$\begin{aligned}{}&u_{r}=[(B+\sum \limits _{j = 1}^{{N_2}} {{B_j}})^T (B+\sum \limits _{j = 1}^{{N_2}} {{B_j}})]^{-1} (B+\sum \limits _{j = 1}^{{N_2}} {{B_j}})^T[(I-A-\sum \limits _{i = 1}^{{N_1}} {{A_i}} )]x_r. \end{aligned}$$

Given the actual and ideal distributions defined in (12–14), the optimal randomised controller of the considered class of linear and Gaussian stochastic systems with multiple state and control input delays can be calculated following Theorem 2. This leads to the randomised control solution specified by the following theorem,

### Theorem 3

By substituting the ideal distribution of the system dynamics (13), the ideal distribution of the controller (14), and the actual distributions of the system dynamics (12) into Eqs. (8–10), the optimal randomised controller $c(u_t|x_{t-1}, x_{t-{h_1}}, \dots , x_{t-{h_{N_1}}}, u_{t-{L_1}}, \dots , u_{t-{L_{N_2}}})$ which minimizes the optimal cost-to-go function (6) is given by,

16 $$\begin{aligned} c(u_t|x_{t-1}, x_{t-{h_1}}, \dots , x_{t-{h_{N_1}}}, u_{t-{L_1}}, \dots , u_{t-{L_{N_2}}}) \sim N(u^*_t, \Gamma _t), \end{aligned}$$

where,

17 $$\begin{aligned} u_t^*&= - {\Gamma _t}{B^T}{{\bar{M}}^T}_tA{x_{t - 1}} - {d_t},\nonumber \\ {d_t}&= {\Gamma _t}[{B^T}{{\bar{M}}^T}_t{f_t} + 0.5{B^T}P_t^T - {B^T}{R^{ - 1}}{x_r} + \rho _{2,t} - {\Gamma ^{ - 1}}{u_{r}}],\nonumber \\ \rho _{2,t}&= 0.5\sum \limits _{j = 1}^{{N_2}} B_j^T\bigg [{M_{t + {L_j}}}\bar{\mu }_{t+L_j} + P^T_{t + {L_j}}\bigg ]\delta (t+L_j),\nonumber \\ \rho _{1,t}&= 0.5\sum _{i=1}^{N_1} (P_{t+h_i} + \bar{\mu }^T_{t+h_i}M_{t+h_i} )A_i \delta (t+h_i), \nonumber \\ f_t&= \sum \limits _{i = 1}^{{N_1}} {{A_i}{x_{t - {h_i} - 1}}} + \sum \limits _{j = 1}^{{N_2}} {{B_j}{u_{t - {L_j}}}}, \nonumber \\ \bar{M}_t&={M_t} + {R^{ - 1}},\nonumber \\ \Gamma _{t}&=\bigg ({B^T}\bar{M}_t B + \Gamma ^{-1}\bigg )^{-1},\nonumber \\ \bar{\mu }_{t+L_j}&=A{x_{t + {L_j} - 1}} + \sum \limits _{i = 1}^{{N_1}} {{A_i}{x_{t + {L_j} - {h_i} - 1}}} + \sum \limits _{d = 1}^{{N_2}} {{B_d}{u_{t + {L_j} - {L_d}}}} + B{{\bar{u}}_{t + {L_j}}},\nonumber \\ \bar{\mu }_{t+h_i}&=A{x_{t + {h_i} - 1}} + \sum \limits _{q = 1}^{{N_1}} {{A_q}{x_{t + {h_i} - {h_q} - 1}}} + \sum \limits _{j = 1}^{{N_2}} {{B_j}{u_{t + {h_i} - {L_j}}}} + B{{\bar{u}}_{t + {h_i}}}, \end{aligned}$$

and where,

18 $$\begin{aligned} - \ln \left( {\gamma \left( {{x_{t - 1}}} \right) } \right) = 0.5x_{t - 1}^T{M_{t - 1}}{x_{t - 1}} + 0.5{P_{t - 1}}{x_{t - 1}} + 0.5{\omega _{t - 1}} , \end{aligned}$$

with,

19 $$\begin{aligned}{}&M_{t-1}= A^T\bigg [-\bar{M}_t B\Gamma _{t}B^T\bar{M}_t+ \bar{M}_t\bigg ] A, \end{aligned}$$

20 $$\begin{aligned}{}&P_{t-1}=-2\bigg ( f^T_t\bar{M}_t B + 0.5{P_t}B - x_r^T{R^{ - 1}}B + \rho ^T_{2,t} -u^T_{r;t}\Gamma ^{-1}\bigg )\Gamma _{t} B^T\bar{M}_tA+ 2f^T_t\bar{M}_tA + 2\rho _{1,t} -2(x_r^T{R^{ - 1}} - 0.5{P_t})A, \end{aligned}$$

and ${\omega _{t - 1}}$ is constant that does not depend on $x_{t-1}$ or $u_t$,

21 $$\begin{aligned}{}&{\omega _{t - 1}} =-2\bigg \{0.5\bigg [{ f^T_t}({M_t} + {R^{ - 1}})B + 0.5{P_t}B - x_r^T{R^{ - 1}}B + \rho ^T_{2,t} -u^T_{r;t}\Gamma ^{-1}\bigg ]^T\bigg ({B^T}({M_t} + {R^{ - 1}})B + \Gamma ^{-1}\bigg )^{-1}\nonumber \\&\times \bigg [{f^T_t}({M_t} + {R^{ - 1}})B + 0.5{P_t}B - x_r^T{R^{ - 1}}B + \rho ^T_{2,t} -u^T_{r;t}\Gamma ^{-1}\bigg ] - 0.5f^T_t({R^{ - 1}} + {M_t})f^T_t -0.5u^T_{r;t}\Gamma ^{-1}u_{r} \nonumber \\&+ (x_r^T{R^{ - 1}} - 0.5{P_t})(A{x_{t - 1}} + f_t ) - 0.5{\omega _t} - 0.5tr({M_t}Q) - 0.5x_r^T{R^{ - 1}}{x_r} + 0.5tr(Q({Q^{ - 1}} - {R^{ - 1}}))\nonumber \\&- \sum \limits _{i = 1}^{{N_1}}\bigg [ 0.5\bar{\mu }^T_{t + {h_i}}{M_{t + {h_i}}}(A{x_{t + {h_i} - 1}} + \sum \limits _{q = 1,h_i\ne h_q}^{{N_1}} {{A_q}{x_{t + {h_i} - {h_q} - 1}}} + B{{\bar{u}}_{t + {h_i}}} + \sum \limits _{j = 1}^{{N_2}} {{B_j}{u_{t + {h_i} - {L_j}}}} )+ 0.5{\omega _{t + {h_i}}} + 0.5tr({M_{t + {h_i}}}Q) \nonumber \\&+ 0.5tr({M_{t + {h_i}}}{\Sigma _{t + {h_i}}})+ 0.5{P_{t + {h_i}}}(A{x_{t + {h_i} - 1}} + \sum \limits _{q = 1,h_i\ne h_q}^{{N_1}} {{A_q}{x_{t + {h_i} - {h_q} - 1}}} + B{{\bar{u}}_{t + {h_i}}} + \sum \limits _{j = 1}^{{N_2}} {{B_j}{u_{t + {h_i} - {L_j}}}} )\bigg ]\delta (t+h_i)\nonumber \\&- \sum \limits _{j = 1}^{{N_2}} \bigg [ 0.5{(A{x_{t + {L_j} - 1}} +B{{\bar{u}}_{t + {L_j}}}+ \sum \limits _{i = 1}^{{N_1}} {{A_i}{x_{t + {L_j} - {h_i} - 1}}} + \sum \limits _{d = 1,{L_d} \ne {L_j}}^{{N_2}} {{B_d}{u_{t + {L_j} - {L_d}}}} )^T}{M_{t + {L_j}}}\bar{\mu } _{t + {L_j}} \nonumber \\&+ 0.5{P_{t + {L_j}}}(A{x_{t + {L_j} - 1}} +B{{\bar{u}}_{t + {L_j}}}+ \sum \limits _{i = 1}^{{N_1}} {{A_i}{x_{t + {L_j} - {h_i} - 1}}} + \sum \limits _{d = 1,{L_d} \ne {L_j}}^{{N_2}} {{B_d}{u_{t + {L_j} - {L_d}}}} ) + 0.5{\omega _{t + {L_j}}} + 0.5tr({\Sigma _{t + {L_j}}}{B^T}{M_{t + {L_j}}}B)\bigg ]\delta (t+L_j) \bigg \}\bigg \}. \end{aligned}$$

### Proof

The proof of this theorem is given in the Supplementary Appendix. $\square$

### Remark 1

Compared to the conventional randomised FPD controller, the mean of the derived randomised controller of the MTDFPC method has an extra linear term $d_t$, so does the Riccati Eq. (18) has an extra linear term $0.5{P_{t - 1}}{x_{t - 1}}$. This is the consequence of the presence of the multiple lagged system state and control input. Besides, the solutions of the Riccati equation as well as the additional linear term in the mean of the optimised control input are dependent upon the delayed and future state and control input.

As has been seen from Eqs. (17–21), both of the solutions of the optimal cost-to-go function and the optimal randomised controller require knowledge of future state and control input values, thus bringing challenges to solve the problem analytically. To address this issue, the successive approximation approach (SAA) introduced in^35^, will be applied in this paper to obtain the numerical solution of the MTDFPC method. The numerical solution will be discussed in the next section.

## Numerical solution to the MTDFPC using SAA

In this section, the SAA will be implemented to obtain the numerical solution of the MTDFPC problem. Proposed in^35^, this approach is developed by iteratively solving a sequence of the corresponding non-homogeneous linear equations in the LQ control problem where each sequence is worked out as a standard numerical problem. Following this approach, the future terms in the optimal control law and corresponding optimality equations can be obtained from previous iterations, thus overcoming the requirement of predicting these future values. For more details about the SAA, the readers are referred^35^. The procedure of a slight variation of the SAA for obtaining the approximate numerical solution of the optimal randomised controller is given in Algorithm 1. As can be seen from this algorithm, the optimisation problem of the randomised controller needs to be done through a number of iterations, *K* where in each iteration the sequence of randomised control inputs that optimises the control objective is obtained from time zero to the final time. Once the first iteration is completed the required future state and control input values can be obtained and used in the next iteration. This process continues until a convergence is achieved. 
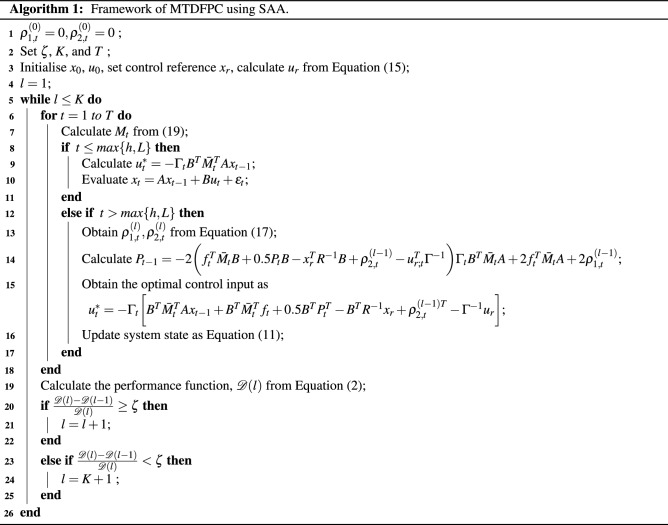


## Experiment

In this section, the proposed control algorithm is tested on one numerical simulation and one practically related simulation to demonstrate the effectiveness of the proposed control method.

### Numerical example

Consider the following three-order delay stochastic system,

22 $$\begin{aligned} {x_t} = A{x_{t - 1}} + {{A_1}{x_{t - {h_1}-1}}} + {{A_2}{x_{t - {h_2}-1}}} + B{u_t} + {{B_1}{u_{t - {L_1}}}} + {{B_2}{u_{t - {L_2}}}} +\varepsilon _t , \end{aligned}$$

where, $$A = \left[ {\begin{array}{*{20}{c}} {0.9}&{}{0.02}&{}0\\ 0&{}{0.92}&{}{0.01}\\ { - 0.05}&{}{ - 0.52}&{}{0.75} \end{array}} \right]$$, $$B = \left[ {\begin{array}{*{20}{c}} 0\\ 0\\ {0.1} \end{array}} \right]$$, $${A_1} = \left[ {\begin{array}{*{20}{c}} {0.01}&{}{0.004}&{}{0.01}\\ { - 0.1}&{}{ - 0.04}&{}{ - 0.01}\\ { - 0.06}&{}{ - 0.01}&{}{0.02} \end{array}} \right]$$, $${B_1} = \left[ {\begin{array}{*{20}{c}} 0\\ 0\\ {0.1} \end{array}} \right]$$,

$${A_2} = \left[ {\begin{array}{*{20}{c}} {0.03}&{}{0.006}&{}{0.01}\\ { - 0.03}&{}{ - 0.02}&{}{ - 0.01}\\ { - 0.04}&{}{ - 0.03}&{}{0.02} \end{array}} \right]$$, $${B_2} = \left[ {\begin{array}{*{20}{c}} 0\\ 0\\ {0.08} \end{array}} \right]$$.

In addition, $\varepsilon _t$ is Gaussian noise with the following distribution $\varepsilon _t \sim N(0,0.02I_{3\times 3})$, where $I_{3 \times 3}$ is the identity matrix of size 3. This example was used in^33^ to demonstrate their theoretical development of LQRs for systems with multiple input and state delays.

In this simulation study, the input time delays are set as $L_1=15$ , $L_2=20$ while the state delays are $h_1=15$, $h_2=12$. Moreover, the initial state vales are given as $x_0=[0.6;-1.5;3]$, $x_t=[0,0,0]^T, t=-max\{h_1,h_2\},-max\{h_1,h_2\}+1,...,-1$ while $u_t=0, t=-max\{L_1,L_2\},-max\{L_1,L_2\}+1,...,1$. The reference state for the system to track is given as $[0.06;-0.1;0.4]$. Furthermore, the SAA control loop is set to be $K=10$.

**Figure 1:**
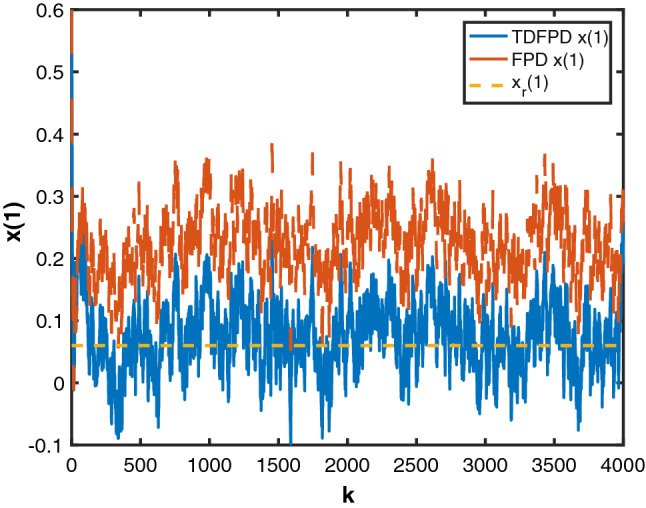
State $x_1$ by MTDFPC, State $x_1$ by FPD and reference $x_r(1)$.

**Figure 2:**
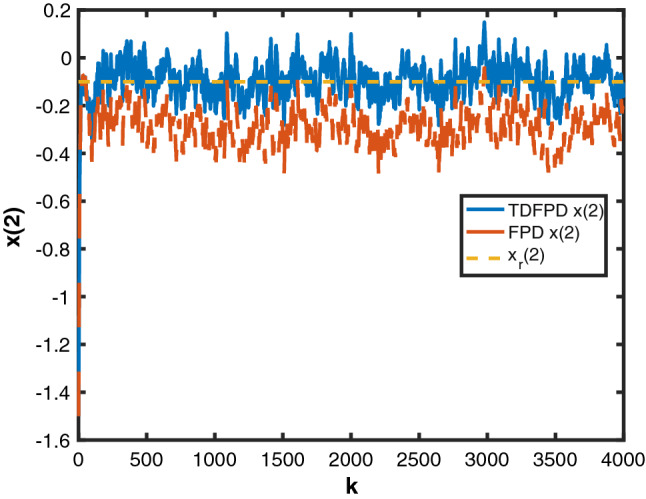
State $x_2$ by MTDFPC, State $x_2$ by FPD and reference $x_r(2)$.

**Figure 3:**
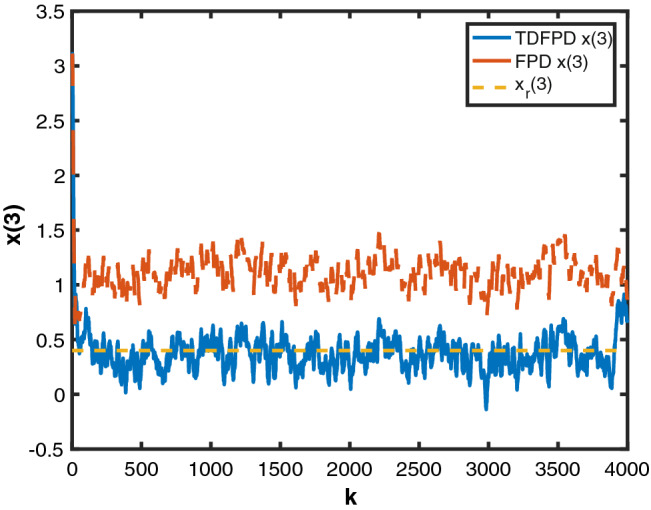
State $x_3$ by MTDFPC, State $x_3$ by FPD and reference $x_r(3)$.

To validate the performance of the MTDFPC derived in this paper, the results are compared to the results obtained from the traditional FPD. The simulation results are given in Figs. 1, 2 and 3, where the blue solid line represents the state responses controlled by MTDFPC, the red dashed line is the state controlled by traditional FPD, and the yellow dotted line stands for their corresponding state references. From these figures, it can be seen that compared with the system state controlled by traditional FPD ,the state controlled by MTDFPC can always track their corresponding reference states, even with the presence of the noise and the multiple state and input delays. On the contrary, the state controlled by the traditional FPD shows large tracking errors. Based on the above results, it can be concluded that compared with traditional FPD method, the proposed MTDFPC algorithm can achieve a much better tracking performance aiming at stochastic systems that are affected by multiple state and control input delays.

### Electric heater system

**Figure 4:**
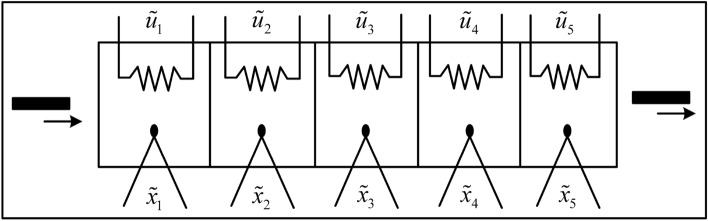
Structure diagram of electric heater.

To demonstrate the effectiveness of the proposed MTDFPC algorithm on real world systems, this section discusses the results of the implementation of the proposed algorithm to an industrial electric heater model which was used in^36,37^. The system structure is given in Fig. 4. From Fig. 4, it can be seen that the heater involves five heating zones, each equipped with an electric heater and their own thermocouple to measure its temperature profile. The system state are the temperatures in each zone which are donated by $\tilde{x}_1,...,\tilde{x}_5$ while control inputs are the electrical current signals applied to each zone of the heater which are donated by $\tilde{u}_1,...,\tilde{u}_5$. The control objective is to maintain the temperature profile of the process at their pre-set operating points $\bar{x}_1$ to $\bar{x}_5$.

A state-delayed nominal discrete tracking-error based model for this system was obtained in^37^ in the following form,

23 $$\begin{aligned} x_{t}=Ax_{t-1} + A_1x_{t-d} + Bu_{t} + \varepsilon _{t}, \ \end{aligned}$$

where the system matrices are given by,

24 $$\begin{aligned} A&= \left[ {\begin{array}{*{20}{c}} {0.97421}&{}{0.15116}&{}{0.19667}&{}{ - 0.0587}&{} 0.07144\\ { - 0.01455}&{}{0.88914}&{}{0.26953}&{}{0.11866} &{} -0.22047\\ {0.06376}&{}{0.12056}&{}{1.0049}&{}{ - 0.03491} &{} -0.02766\\ {-0.05084}&{}{0.09254}&{}{0.28774}&{}{0.82569}&{}0.02570 \\ 0.01723 &{} 0.01939 &{} 0.29285 &{}0.03544 &{} 0.87111 \end{array}} \right] ,\nonumber \\ A_1&= \left[ {\begin{array}{*{20}{c}} {-0.01000}&{}{-0.08837}&{}{-0.06989}&{}{0.18874}&{} 0.20505\\ { 0.02363}&{}{0.03384}&{}{0.05282}&{}{-0.09906} &{} -0.00191\\ {-0.04468}&{}{-0.00798}&{}{0.05618}&{}{ 0.00157} &{} 0.03593\\ {-0.04082}&{}{0.01153}&{}{-0.07116}&{}{0.16472}&{} 0.00083 \\ -0.02527 &{} 0.03878 &{} -0.04683 &{}0.05665 &{} -0.03130 \end{array}} \right] ,\nonumber \\ B&= \left[ {\begin{array}{*{20}{c}} {0.53706}&{}{-0.11185}&{}{0.09978}&{}{0.04652}&{} 0.25867\\ { - 0.51718}&{}{0.73519}&{}{0.57518}&{}{0.40668} &{} -0.12472\\ {0.29469}&{}{0.31528}&{}{1.16420}&{}{ -0.29922} &{} 0.23883\\ {-0.20191}&{}{0.19739}&{}{0.41686}&{}{0.66551}&{} 0.11366 \\ -0.11835 &{} 0.16287 &{} 0.20378 &{}0.23261 &{} 0.36525 \end{array}} \right] . \end{aligned}$$

In addition, the state and control vectors are defined as,

25 $$\begin{aligned} x&=[\tilde{x}_1-\bar{x}_1,\tilde{x}_2-\bar{x}_2,\tilde{x}_3-\bar{x}_3,\tilde{x}_4-\bar{x}_4,\tilde{x}_5-\bar{x}_5 ]^T ,\nonumber \\ u&=[\tilde{u}_1-\bar{u}_1,\tilde{u}_2-\bar{u}_2,\tilde{u}_3-\bar{u}_3,\tilde{u}_4-\bar{u}_4,\tilde{u}_5-\bar{u}_5 ]^T, \end{aligned}$$

where $\tilde{x}_1$ to $\tilde{x}_5$ are each heater’s temperature as introduced and $\bar{x}_1$ to $\bar{x}_5$ represent their operating points that they need to follow, similarly, $\tilde{u}_1$ to $\tilde{u}_5$ stand for the control electric current in each zone and $\bar{u}_1$ to $\bar{u}_5$ are their corresponding operating points. The system state $x_t$ in Eq. (23) is the tracking error of each zone, indicating that the control objective here is to make sure the system state stays around zero. Moreover, $\varepsilon _t$ is Gaussian noise representing the uncertainties and disturbance that the system is affected by. The distribution of $\varepsilon _t$ is given as follows,

26 $$\begin{aligned} \varepsilon _t \sim N(0,0.03I_{5 \times 5}), \end{aligned}$$

where $I_{5\times 5}$ is the identity matrix of size 5. In this simulation study, the state delay is taken to be $d=15$ while the initial value of state is taken to be $x_0=[-0.2, 0.5 , 1 ,-0.4, 0.9]^T$, and $x_t=[0,0,0,0,0]^T, t=-d+1,...,-1$. The SAA control loop is set to be $K=3$. As introduced earlier, the reference state are zero in this case, $x_r=[0,0,0,0,0]^T$.

**Figure 5:**
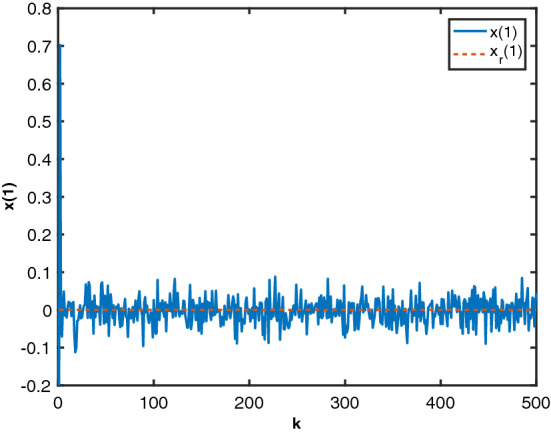
State $x_1$ and reference $x_r(1)$.

**Figure 6:**
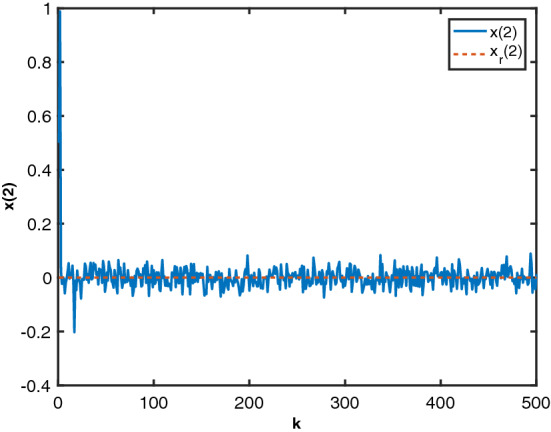
State $x_2$ and reference $x_r(2)$.

**Figure 7:**
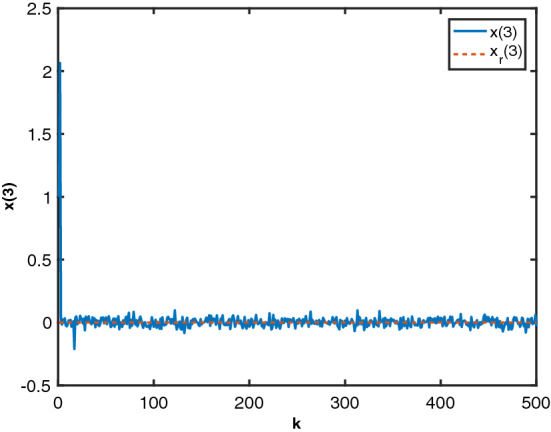
State $x_3$ and reference $x_r(3)$.

**Figure 8:**
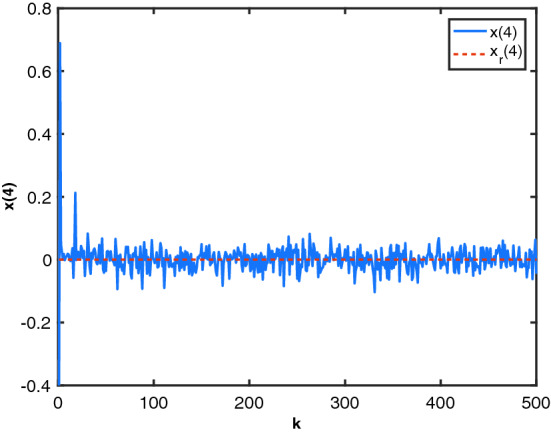
State $x_4$ and reference $x_r(4)$.

**Figure 9:**
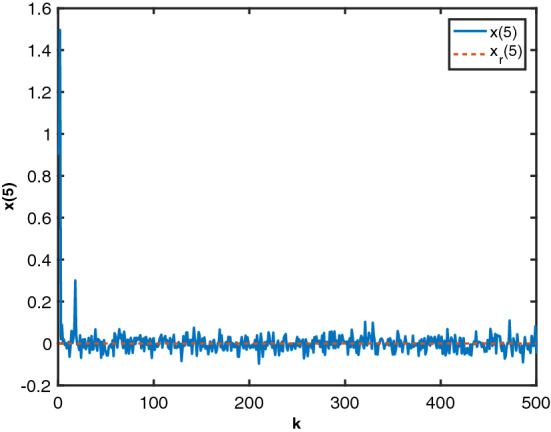
State $x_5$ and reference $x_r(5)$.

Following the procedure provided in Algorithm I, the system state response are given in Figs. 5, 6, 7, 8 and 9. From these figures, we can see that despite the influence of the noise and the state delays, the designed local randomised controllers have successfully brought all the states to zero, indicating that all the heaters’ temperatures are following their operating points. The results illustrate that the proposed algorithm achieved a satisfactory performance for the electric heater system that involves noises and state delays.

## Conclusion

In this paper, the optimal randomised control problem for stochastic discrete-time systems that are affected by multiple control input and state delays has been considered. Probabilistic state-space models are exploited to characterise the dynamics of the system and a MTDFPC control framework is developed by considering the multiple delays of the system state and control input into the derivation of the optimal randomised controller. Moreover, the analytic optimal control law for a class of linear Gaussian stochastic systems is obtained and its numerical solution is evaluated using the SAA method. Finally, one numerical example and one practical example demonstrated the effectiveness of the proposed MTDFPC framework for stochastic systems that are affected by multiple delays and randomness.

## Supplementary Information

Supplementary Information.

## References

[1] Niculescu SI (2001). Delay Effects on Stability: A Robust Control Approach.

[2] Sykora HT, Sadeghpour M, Ge JI, Bachrathy D, Orosz G (2020). On the moment dynamicsof stochastically delayed linear control systems. Int. J. Robust Nonlinear Control.

[3] Lian FL, Moyne JR, Tilbury DM (2001). Performance evaluation of control networks: Ethernet, controlnet, and devicenet. IEEE Control Syst. Mag..

[4] Smith O (1957). Closer control of loops with dead time. Chem. Eng. Prog..

[5] Watanabe K, Ito M (1981). A process-model control for linear systems with delay. IEEE Trans. Autom. Control.

[6] Gumussoy S, Michiels W (2011). Fixed-order h-infinity control for interconnected systems using delay differential algebraic equations. SIAM J. Control Optim..

[7] Özbay HGS (2008). Stable controller design for time-delay system. Int. J. Control.

[8] Zope, R., Mohammadpour, J., Grigoriadis, K. & Franchek, M. Delay-dependent h$$_\infty$$ control for lpv systems with fast-varying time delays. in American Control Conference (Fairmont Queen Elizabeth, Montréal, Canada, 2012).

[9] Shen S , Song A, Li T (2019). Predictor-based motion tracking control for cloud robotic systems with delayed measurements. Electronics.

[10] Zhang Y (2019). Analysis methodology for evaluation of time-delay impact on network-based system for droop-controlled ac microgrid. Electronics.

[11] Keyvan-Ekbatani M, Papageorgiou M, Knoop VL (2015). Controller design for gating traffic control in presence of time-delay in urban road networks. Transp. Res. Proc..

[12] Guo P, Zhang J, Lyu M, Bo Y (2013). Sliding mode control for multiagent system with time-delay and uncertainties: An lmi approach. Math. Probl. Eng..

[13] Wernecke H, Sándor B, Gros C (2019). Chaos in time delay systems, an educational review. Phys. Rep..

[14] Gu W, Yu Y, Hu W (2017). Artificial bee colony algorithm based parameter estimation of fractional-order chaotic system with time delay. IEEE/CAA J. Autom. Sin..

[15] Lakshmanan M, Senthilkumar DV (2011). Dynamics of Nonlinear Time-delay Systems.

[16] Si-Ammour A, Djennoune S, Bettayeb M (2009). A sliding mode control for linear fractional systems with input and state delays. Commun. Nonlinear Sci. Numer. Simul..

[17] Bhalekar S, Daftardar-Gejji V (2011). A predictor-corrector scheme for solving nonlinear delay differential equations of fractional order. J. Fract. Calc. Appl..

[18] Sönmez S, Ayasun S (2015). Stability region in the parameter space of pi controller for a single-area load frequency control system with time delay. IEEE Trans. Power Syst..

[19] Pradhan SK, Das DK (2020). H$$\infty$$ load frequency control design based on delay discretization approach for interconnected power systems with time delay. J. Mod. Power Syst. Clean Energy.

[20] Tan Y, Liu Y, Niu B, Fei S (2020). Event-triggered synchronization control for t-s fuzzy neural networked systems with time delay. J. Franklin Inst..

[21] Gu K , Niculescu SI (2003). Survey on recent results in the stability and control of time-delay systems. J. Dyn. Syst. Meas. Control.

[22] Richard J-P (2003). Time-delay systems: An overview of some recent advances and open problems. Automatica.

[23] Zhang H, Duan G, Xie L (2006). Linear quadratic regulation for linear time-varying systems with multiple input delays. Automatica.

[24] Sykora HT, Bachrathy D, Stepan G (2019). Stochastic semi-discretization for linearstochastic delay differential equations. Int. J. Numer. Meth. Eng..

[25] Song R, Zhu Q (2018). Stability of linear stochastic delay differential equations with infinite markovian switchings. Int. J. Robust Nonlinear Control.

[26] Lu J, Xi Y, Li D (2019). Stochastic model predictive control for probabilistically constrained markovian jump linear systems with additive disturbance. Int. J. Robust Nonlinear Control.

[27] Peters EGW, Marelli D, Quevedo DE, Fu M (2019). Predictive control for networked systems affected by correlated packet loss. Int. J. Robust Nonlinear Control.

[28] Kárnỳ M (1996). Towards fully probabilistic control design. Automatica.

[29] Herzallah R, Kárnỳ M (2011). Fully probabilistic control design in an adaptive critic framework. Neural Netw..

[30] Zhou, Y., Herzallah, R. & Zafar, A. Fully probabilistic design for stochastic discrete system with multiplicative noise. in *booktitle2019 IEEE 15th International Conference on Control and Automation (ICCA)*, 940–945 (organizationIEEE, 2019).

[31] Zhou Y, Herzallah R (2020). Dobc based fully probability design for stochastic system with the multiplicative noise. IEEE Access.

[32] Herzallah R (2020). A fully probabilistic design for stochastic systems with input delay. Int. J. Control.

[33] Wang H-H, Tang G-Y (2009). Observer-based optimal output tracking for discrete-time systems with multiple state and input delays. Int. J. Control Autom. Syst..

[34] Peterka, V. Bayesian Approach To System Identification. in Trends and Progress in System identification, 239–304 (Elsevier, 1981).

[35] Tang G, Wang H (2005). Suboptimal control for discrete linear systems with time-delay: A successive approximation approach. Acta Autom. Sin..

[36] Taşçıkaraoglu FY, Ucun L, Küçükdemiral IB (2015). Receding horizon h$$\infty$$ control of time-delay systems. Trans. Inst. Meas. Control..

[37] Chu J (1995). Application of a discrete optimal tracking controller to an industrial electric heater with pure delays. J. Process Control.

