# Author Correction: A fully probabilistic control framework for stochastic systems with input and state delay

**DOI:** 10.1038/s41598-022-15045-5

**Published:** 2022-06-23

**Authors:** Randa Herzallah, Yuyang Zhou

**Affiliations:** Mathematics Group, Aston University, Aston Triangle, Birmingham, B4 7ET UK

Correction to: *Scientific Reports* 10.1038/s41598-022-11514-z, published online 12 May 2022

The Acknowledgements section in the original version of this Article was incomplete.

“This work was supported by the Leverhulme Trust under Grant RPG-2017-337.”

now reads:

“This work was supported by the Leverhulme Trust under Grant RPG-2017-337. This work was supported by the EPSRC Grant EP/V048074/1.”

The original Article has been corrected.

