# Supplementary material for: A fully probabilistic control framework for stochastic systems with input and state delay
Source: Sci Rep. 2022 May 12;12:7812. doi: 10.1038/s41598-022-11514-z (PMC9098898; doi:10.1038/s41598-022-11514-z)
Supplement: Supplementary file 1 — Supplementary Information. [file 41598_2022_11514_MOESM1_ESM.pdf]

A Fully Probabilistic Control Framework for Stochastic Systems with Input and State Delay
Randa Herzallah and Yuyang Zhou

Appendix

Proof of the Recurrence Functional Equation

The recurrence functional equation given in (6) of the proposed fully probabilistic control for stochastic systems with state and input time delays can be obtained from the stochastic principle of optimality. The stochastic principle of optimality can be derived from the optimal cost to go function defined in Equation (5) repeated here,

$$
\begin{aligned}
-\ln(\gamma(x_{t-1})) = & \min_{c(u_\tau|x_{\tau-1},x_{\tau-h_1},\dots,x_{\tau-h_{N_1}},u_{\tau-L_1},\dots,u_{\tau-L_{N_2}})} \sum_{\tau=t}^{T} \int f(\mathscr{X}_\tau,\dots,\mathscr{X}_T|x_{\tau-1}) \\
& \times \Bigg[\ln\left(\frac{s(x_\tau|x_{\tau-1},u_\tau,x_{\tau-h_1},\dots,x_{\tau-h_{N_1}},u_{\tau-L_1},\dots,u_{\tau-L_{N_2}})}{{}^{I}s(x_\tau|x_{\tau-1},u_\tau,x_{\tau-h_1},\dots,x_{\tau-h_{N_1}},u_{\tau-L_1},\dots,u_{\tau-L_{N_2}})}\right) \\
& + \ln\left(\frac{c(u_\tau|x_{\tau-1},x_{\tau-h_1},\dots,x_{\tau-h_{N_1}},u_{\tau-L_1},\dots,u_{\tau-L_{N_2}})}{{}^{I}c(u_\tau|x_{\tau-1},x_{\tau-h_1},\dots,x_{\tau-h_{N_1}},u_{\tau-L_1},\dots,u_{\tau-L_{N_2}})}\right) \Bigg] \mathrm{d}(\mathscr{X}_\tau,\dots,\mathscr{X}_T).
\end{aligned}
\tag{27}
$$

Note that here the control input, u_t regulates the system by applying the values of u_t at the current time t as well as delayed times $\sum_{j=1}^{N_2} t-L_j$. Similarly, the state is affected by delayed values of the state at delayed times $\sum_{i=1}^{N_1} t-h_i$. Thus, to consider the effect of the delayed control input and states on the future state values, the cost to go function (27) can be explicitly rewritten to consider these delays as follows,

$$
\begin{aligned}
-\ln(\gamma(x_{t-1})) = & \min_{c(u_\tau|x_{t-1},x_{\tau-h_1},\dots,x_{\tau-h_{N_1}},u_{\tau-L_1},\dots,u_{\tau-L_{N_2}})} \\
& \sum_{\tau=t}^{T} \int f(\mathscr{X}_\tau,\dots,\mathscr{X}_T|x_{\tau-1}) \Bigg\{ \ln\left(\frac{s(x_\tau|x_{\tau-1},u_\tau,x_{\tau-h_1},\dots,x_{\tau-h_{N_1}},u_{\tau-L_1},\dots,u_{\tau-L_{N_2}})}{{}^{I}s(x_\tau|x_{\tau-1},u_\tau,x_{\tau-h_1},\dots,x_{\tau-h_{N_1}},u_{\tau-L_1},\dots,u_{\tau-L_{N_2}})}\right) \\
& + \ln\left(\frac{c(u_\tau|x_{\tau-1},x_{\tau-h_1},\dots,x_{\tau-h_{N_1}},u_{\tau-L_1},\dots,u_{\tau-L_{N_2}})}{{}^{I}c(u_\tau|x_{\tau-1},x_{\tau-h_1},\dots,x_{\tau-h_{N_1}},u_{\tau-L_1},\dots,u_{\tau-L_{N_2}})}\right) \\
& + \sum_{i=1}^{N_1} f(\mathscr{X}_{\tau+h_i},\dots,\mathscr{X}_{T+h_i}|x_{\tau-1+h_i}) \sum_{\tau=t+h_i+1}^{T} f(\mathscr{X}_\tau,\dots,\mathscr{X}_T|x_{\tau-1}) \\
& \times \Bigg[\ln\left(\frac{s(x_\tau|x_{\tau-1},u_\tau,x_{\tau-h_1},\dots,x_{\tau-h_{N_1}},u_{\tau-L_1},\dots,u_{\tau-L_{N_2}})}{{}^{I}s(x_\tau|x_{\tau-1},u_\tau,x_{\tau-h_1},\dots,x_{\tau-h_{N_1}},u_{\tau-L_1},\dots,u_{\tau-L_{N_2}})}\right) \\
& + \ln\left(\frac{c(u_\tau|x_{\tau-1},x_{\tau-h_1},\dots,x_{\tau-h_{N_1}},u_{\tau-L_1},\dots,u_{\tau-L_{N_2}})}{{}^{I}c(u_\tau|x_{\tau-1},x_{\tau-h_1},\dots,x_{\tau-h_{N_1}},u_{\tau-L_1},\dots,u_{\tau-L_{N_2}})}\right) \Bigg] \\
& + \sum_{j=1}^{N_2} f(\mathscr{X}_{\tau+L_j},\dots,\mathscr{X}_{T+L_j}|x_{\tau-1+L_j}) \sum_{\tau=t+L_j+1}^{T} f(\mathscr{X}_\tau,\dots,\mathscr{X}_T|x_{\tau-1}) \\
& \times \Bigg[\ln\left(\frac{s(x_\tau|x_{\tau-1},u_\tau,x_{\tau-h_1},\dots,x_{\tau-h_{N_1}},u_{\tau-L_1},\dots,u_{\tau-L_{N_2}})}{{}^{I}s(x_\tau|x_{\tau-1},u_\tau,x_{\tau-h_1},\dots,x_{\tau-h_{N_1}},u_{\tau-L_1},\dots,u_{\tau-L_{N_2}})}\right) \\
& + \ln\left(\frac{c(u_\tau|x_{\tau-1},x_{\tau-h_1},\dots,x_{\tau-h_{N_1}},u_{\tau-L_1},\dots,u_{\tau-L_{N_2}})}{{}^{I}c(u_\tau|x_{\tau-1},x_{\tau-h_1},\dots,x_{\tau-h_{N_1}},u_{\tau-L_1},\dots,u_{\tau-L_{N_2}})}\right) \Bigg] \mathrm{d}(\mathscr{X}_\tau,\dots,\mathscr{X}_T) \Bigg\}.
\end{aligned}
\tag{28}
$$

To re-emphasize, the expanded form in (27) explicitly shows the future cost of the lagged state at time instants $\tau = t+h_i+1$ and lagged control input at time instants $\tau = t+L_j+1$ as represented by the first and second square bracketed terms respectively. Using the dynamic programming principle of optimality, the minimisation of the above equation can be done in two stages. The first stage considers the control input at time t and the second stage considers the control over the remaining period. This can be

represented mathematically as follows,

$$
\begin{aligned}
&-\ln(\gamma(x_{t-1})) = \min_{c(u_t|x_{t-1},x_{t-h_1},\ldots,x_{t-h_{N_1}},u_{t-L_1},\ldots,u_{t-L_{N_2}})} \Bigg\{ \min_{c(u_\tau|x_{t-1},x_{\tau-h_1},\ldots,x_{\tau-h_{N_1}},u_{\tau-L_1},\ldots,u_{\tau-L_{N_2}})_{\tau=t+1}^{T}} \\
&\Bigg\{ \min_{c(u_\tau|x_{t-1},x_{\tau-h_1},\ldots,x_{\tau-h_{N_1}},u_{\tau-L_1},\ldots,u_{\tau-L_{N_2}})_{\tau=t+h_i}^{T}} \Bigg\{ \min_{c(u_\tau|x_{t-1},x_{\tau-h_1},\ldots,x_{\tau-h_{N_1}},u_{\tau-L_1},\ldots,u_{\tau-L_{N_2}})_{\tau=t+L_j}^{T}} \\
&f(\mathscr{X}_1,\ldots,\mathscr{X}_t|x_{t-1})\Bigg\{ \ln\left(\frac{s(x_t|x_{t-1},u_t,x_{t-h_1},\ldots,x_{t-h_{N_1}},u_{t-L_1},\ldots,u_{t-L_{N_2}})}{{}^{I}s(x_t|x_{t-1},u_t,x_{t-h_1},\ldots,x_{t-h_{N_1}},u_{t-L_1},\ldots,u_{t-L_{N_2}})}\right) \\
&+\ln\left(\frac{c(u_t|x_{t-1},x_{t-h_1},\ldots,x_{t-h_{N_1}},u_{t-L_1},\ldots,u_{t-L_{N_2}})}{{}^{I}c(u_t|x_{t-1},x_{t-h_1},\ldots,x_{t-h_{N_1}},u_{t-L_1},\ldots,u_{t-L_{N_2}})}\right) \\
&+\sum_{\tau=t+1}^{T}\int f(\mathscr{X}_\tau,\ldots,\mathscr{X}_T|x_{\tau-1})\ln\left(\frac{s(x_\tau|x_{\tau-1},u_\tau,x_{\tau-h_1},\ldots,x_{\tau-h_{N_1}},u_{\tau-L_1},\ldots,u_{\tau-L_{N_2}})}{{}^{I}s(x_\tau|x_{\tau-1},u_\tau,x_{\tau-h_1},\ldots,x_{\tau-h_{N_1}},u_{\tau-L_1},\ldots,u_{\tau-L_{N_2}})}\right) \\
&+\ln\left(\frac{c(u_\tau|x_{\tau-1},x_{\tau-h_1},\ldots,x_{\tau-h_{N_1}},u_{\tau-L_1},\ldots,u_{\tau-L_{N_2}})}{{}^{I}c(u_\tau|x_{\tau-1},x_{\tau-h_1},\ldots,x_{\tau-h_{N_1}},u_{\tau-L_1},\ldots,u_{\tau-L_{N_2}})}\right) \\
&+\sum_{i=1}^{N_1} f(\mathscr{X}_{\tau+h_i},\ldots,\mathscr{X}_{T+h_i}|x_{\tau-1+h_i}) \sum_{\tau=t+h_i+1}^{T} f(\mathscr{X}_\tau,\ldots,\mathscr{X}_T|x_{\tau-1}) \\
&\times\Bigg[\ln\left(\frac{s(x_\tau|x_{\tau-1},u_\tau,x_{\tau-h_1},\ldots,x_{\tau-h_{N_1}},u_{\tau-L_1},\ldots,u_{\tau-L_{N_2}})}{{}^{I}s(x_\tau|x_{\tau-1},u_\tau,x_{\tau-h_1},\ldots,x_{\tau-h_{N_1}},u_{\tau-L_1},\ldots,u_{\tau-L_{N_2}})}\right) \\
&+\ln\left(\frac{c(u_\tau|x_{\tau-1},x_{\tau-h_1},\ldots,x_{\tau-h_{N_1}},u_{\tau-L_1},\ldots,u_{\tau-L_{N_2}})}{{}^{I}c(u_\tau|x_{\tau-1},x_{\tau-h_1},\ldots,x_{\tau-h_{N_1}},u_{\tau-L_1},\ldots,u_{\tau-L_{N_2}})}\right)\Bigg] \\
&+\sum_{j=1}^{N_2} f(\mathscr{X}_{\tau+L_j},\ldots,\mathscr{X}_{T+L_j}|x_{\tau-1+L_j}) \sum_{\tau=t+L_j+1}^{T} f(\mathscr{X}_\tau,\ldots,\mathscr{X}_T|x_{\tau-1}) \\
&\times\Bigg[\ln\left(\frac{s(x_\tau|x_{\tau-1},u_\tau,x_{\tau-h_1},\ldots,x_{\tau-h_{N_1}},u_{\tau-L_1},\ldots,u_{\tau-L_{N_2}})}{{}^{I}s(x_\tau|x_{\tau-1},u_\tau,x_{\tau-h_1},\ldots,x_{\tau-h_{N_1}},u_{\tau-L_1},\ldots,u_{\tau-L_{N_2}})}\right) \\
&+\ln\left(\frac{c(u_\tau|x_{\tau-1},x_{\tau-h_1},\ldots,x_{\tau-h_{N_1}},u_{\tau-L_1},\ldots,u_{\tau-L_{N_2}})}{{}^{I}c(u_\tau|x_{\tau-1},x_{\tau-h_1},\ldots,x_{\tau-h_{N_1}},u_{\tau-L_1},\ldots,u_{\tau-L_{N_2}})}\right)\Bigg]\Bigg\}\mathrm{d}(\mathscr{X}_\tau,\ldots,\mathscr{X}_T)\Bigg\}\Bigg\}\Bigg\}.
\end{aligned}
\tag{29}
$$

Using the definition of the minimum cost-to-go function given in (27), respecting the dependence of the individual terms on the optimised control input, and noting that the next to the last and the last square bracketed terms are zero when $t = T-h_i, T-h_i+1,\ldots$ and $t = T-L_j, T-L_j+1,\ldots$ respectively, equation (29) simplifies to give Equation (6). This completes the proof.

Proof of Theorem 2

The derivation of Equation (8) can be reached by evaluating the recurrence equation defined in Equation (6). Using Fubini's theorem, the definitions given in Equation (9) and (10) can be obtained. Substituting Equation (10) in Equation (6) yields,

$$
\begin{aligned}
&-\ln(\gamma(x_{t-1})) = \min_{c(u_t|x_{t-1},x_{t-h_1},\ldots,x_{t-h_{N_1}},u_{t-L_1},\ldots,u_{t-L_{N_2}})} \int c(u_t|x_{t-1},x_{t-h_1},\ldots,x_{t-h_{N_1}},u_{t-L_1},\ldots,u_{t-L_{N_2}}) \\
&\Bigg[\beta_1(.)+\beta_2(.)+\int c(u_{t+h_i}|x_{t+h_i-1},x_{t+h_i-h_1},\ldots,x_{t+h_i-h_{N_1}},u_{t+h_i-L_1},\ldots,u_{t+h_i-L_{N_2}})\beta_3(.)\delta(t+h_i)\mathrm{d}u_{t+h_i} \\
&+\int c(u_{t+L_j}|x_{t+L_j-1},x_{t+L_j-h_1},\ldots,x_{t+L_j-h_{N_1}},u_{t+L_j-L_1},\ldots,u_{t+L_j-L_{N_2}})\beta_4(.)\delta(t+L_j)\mathrm{d}u_{t+L_j} \\
&+\ln\frac{c(u_t|x_{t-1},x_{t-h_1},\ldots,x_{t-h_{N_1}},u_{t-L_1},\ldots,u_{t-L_{N_2}})}{{}^{I}c(u_t|x_{t-1},x_{t-h_1},\ldots,x_{t-h_{N_1}},u_{t-L_1},\ldots,u_{t-L_{N_2}})}\Bigg]\mathrm{d}u_t.
\end{aligned}
\tag{30}
$$

Using Equation (9) in the above equation yields,

$$-\ln(\gamma(x_{t-1})) = \min_{c(u_t|x_{t-1},x_{t-h_1},\ldots,x_{t-h_{N_1}},u_{t-L_1},\ldots,u_{t-L_{N_2}})} \int c(u_t|x_{t-1},x_{t-h_1},\ldots,x_{t-h_{N_1}},u_{t-L_1},\ldots,u_{t-L_{N_2}})$$

$$\left[\beta_1(.)+\beta_2(.)+\tilde{\beta}_3(.)+\tilde{\beta}_4(.)+\ln\frac{c(u_t|x_{t-1},x_{t-h_1},\ldots,x_{t-h_{N_1}},u_{t-L_1},\ldots,u_{t-L_{N_2}})}{{}^I c(u_t|x_{t-1},x_{t-h_1},\ldots,x_{t-h_{N_1}},u_{t-L_1},\ldots,u_{t-L_{N_2}})}\right]\mathrm{d}u_t.$$

$$= \min_{c(u_t|x_{t-1},x_{t-h_1},\ldots,x_{t-h_{N_1}},u_{t-L_1},\ldots,u_{t-L_{N_2}})} \int c(u_t|x_{t-1},x_{t-h_1},\ldots,x_{t-h_{N_1}},u_{t-L_1},\ldots,u_{t-L_{N_2}})$$

$$\left[\ln\frac{c(u_t|x_{t-1},x_{t-h_1},\ldots,x_{t-h_{N_1}},u_{t-L_1},\ldots,u_{t-L_{N_2}})}{\frac{{}^I c(u_t|x_{t-1},x_{t-h_1},\ldots,x_{t-h_{N_1}},u_{t-L_1},\ldots,u_{t-L_{N_2}})\exp[-\beta_1(.)-\beta_2(.)-\tilde{\beta}_3(.)-\tilde{\beta}_4(.)]}{-\ln(\gamma(x_{t-1}))}} - \ln(\gamma(x_{t-1}))\right]\mathrm{d}u_t. \tag{31}$$

The first term is the conditional KLD between the control input distribution and its optimal one, which is minimized for equal arguments and it is zero when the equality is achieved. This completes the proof.

Proof of Theorem 3

To evaluate the optimal randomised controller $c(u_t|x_{t-1},x_{t-h_1},\ldots,x_{t-h_{N_1}},u_{t-L_1},\ldots,u_{t-L_{N_2}})$, Equations (8)–(10) given in Theorem 2 need to be evaluated. According to Equations (8), (9) and (10), the first step is to calculate β_1 to β_4. Using the first equation in (10), β_1 is evaluated as follows,

$$\beta_1(.) = \int \exp\Big[-0.5(x_t-\mu_t)^T Q^{-1}(x_t-\mu_t)\Big]\Big[-0.5(x_t-\mu_t)^T Q^{-1}(x_t-\mu_t)+0.5(x_t-x_r)^T R^{-1}(x_t-x_r)\Big]\mathrm{d}x_t$$
$$= 0.5(Ax_{t-1}+\sum_{i=1}^{N_1}A_i x_{t-h_i-1}+\sum_{j=1}^{N_2}B_j u_{t-L_j})^T R^{-1}(Ax_{t-1}+\sum_{i=1}^{N_1}A_i x_{t-h_i-1}+\sum_{j=1}^{N_2}B_j u_{t-L_j})$$
$$+0.5u_t^T B^T R^{-1}Bu_t + [(Ax_{t-1}+\sum_{i=1}^{N_1}A_i x_{t-h_i-1}+\sum_{j=1}^{N_2}B_j u_{t-L_j})^T R^{-1}B - x_r^T R^{-1}B]u_t$$
$$-x_r^T R^{-1}(Ax_{t-1}+\sum_{i=1}^{N_1}A_i x_{t-h_i-1}+\sum_{j=1}^{N_2}B_j u_{t-L_j})+0.5x_r^T R^{-1}x_r - 0.5tr(Q(Q^{-1}-R^{-1})), \tag{32}$$

where we used equations (12) and (13) to evaluate the integral. Using the second equation in (10), β_2 is evaluated as follows,

$$\beta_2(.) = \int \exp\Big[-0.5(x_t-\mu_t)^T Q^{-1}(x_t-\mu_t)\Big]\Big[0.5x_t^T M_t x_t + 0.5P_t x_t + 0.5\omega_t\Big]\mathrm{d}x_t$$
$$= 0.5\mu_t^T M_t \mu_t + 0.5P_t\mu_t + 0.5\omega_t + 0.5tr(M_t Q)$$
$$= 0.5(Ax_{t-1}+\sum_{i=1}^{N_1}A_i x_{t-h_i-1}+\sum_{j=1}^{N_2}B_j u_{t-L_j})^T M_t(Ax_{t-1}+\sum_{i=1}^{N_1}A_i x_{t-h_i-1}+\sum_{j=1}^{N_2}B_j u_{t-L_j})$$
$$+0.5u_t^T B^T M_t B u_t + [(Ax_{t-1}+\sum_{i=1}^{N_1}A_i x_{t-h_i-1}+\sum_{j=1}^{N_2}B_j u_{t-L_j})^T M_t B$$
$$+0.5P_t B]u_t + 0.5P_t(Ax_{t-1}+\sum_{i=1}^{N_1}A_i x_{t-h_i-1}+\sum_{j=1}^{N_2}B_j u_{t-L_j}) + 0.5\omega_t + 0.5tr(M_t Q), \tag{33}$$

where we used the form of $\gamma(x_t)$ that is defined in equation (18) to evaluate the integral. To evaluate β_3 the third equation in (10) is used to give,

$$\beta_3(.) = \sum_{i=1}^{N_1}\int \exp\Big[-0.5(x_{t+h_i}-\mu_{t+h_i})^T Q^{-1}(x_{t+h_i}-\mu_{t+h_i})-0.5(x_t-\mu_t)^T Q^{-1}(x_t-\mu_t)\Big]$$
$$\times\Big[0.5x_{t+h_i}^T M_{t+h_i}x_{t+h_i}+0.5P_{t+h_i}x_{t+h_i}+0.5\omega_{t+h_i}\Big]\mathrm{d}(x_t,x_{t+h_i})$$
$$= \sum_{i=1}^{N_1} 0.5\mu_{t+h_i}^T M_{t+h_i}\mu_{t+h_i}+0.5P_{t+h_i}\mu_{t+h_i}+0.5\omega_{t+h_i}, \tag{34}$$

where we used the form of $\gamma(x_{t+h_i})$ that is shown in equation (18) to evaluate the integral. Finally β_4 is evaluated using the last equation in (10) as follows,

$$\beta_4(.) = \sum_{j=1}^{N_2} \int \exp\Big[-0.5(x_{t+L_j}-\mu_{t+L_j})^T Q^{-1}(x_{t+L_j}-\mu_{t+L_j}) - 0.5(x_t-\mu_t)^T Q^{-1}(x_t-\mu_t)\Big]$$
$$\times \Big[0.5x^T_{t+L_j}M_{t+L_j}x_{t+L_j} + 0.5P_{t+L_j}x_{t+L_j} + 0.5\omega_{t+L_j}\Big]\mathrm{d}(x_t, x_{t+L_j})$$
$$= \sum_{j=1}^{N_2} 0.5\mu^T_{t+L_j}M_{t+L_j}\mu_{t+L_j} + 0.5P_{t+L_j}\mu_{t+L_j} + 0.5\omega_{t+L_j}, \tag{35}$$

where we used the form of $\gamma(x_{t+L_j})$ that is defined in equation (18) to evaluate the integral. Then, using $\beta_3(.)$ as obtained from (34) in the second equation in (10), $\tilde{\beta}_3(.)$ can be specified as follows,

$$\tilde{\beta}_3(.) = \int c(u_{t+h_i}|x_{t+h_i-1}, x_{t+h_i-h_1}, \dots, x_{t+h_i-h_{N_1}}, u_{t+h_i-L_1}, \dots, u_{t+h_i-L_{N_2}})\beta_3(.)\delta(t+h_i)\mathrm{d}u_{t+h_i}$$
$$= \sum_{i=1}^{N_1} \int c(u_{t+h_i}|x_{t+h_i-1}, x_{t+h_i-h_1}, \dots, x_{t+h_i-h_{N_1}}, u_{t+h_i-L_1}, \dots, u_{t+h_i-L_{N_2}})\Big[0.5(Ax_{t+h_i-1}$$
$$+ \sum_{q=1}^{N_1} A_q x_{t+h_i-h_q-1} + \sum_{j=1}^{N_2} B_j u_{t+h_i-L_j})^T M_{t+h_i}(Ax_{t+h_i-1} + \sum_{q=1}^{N_1} A_q x_{t+h_i-h_q-1} + \sum_{j=1}^{N_2} B_j u_{t+h_i-L_j})$$
$$+ (Ax_{t+h_i-1} + \sum_{q=1}^{N_1} A_q x_{t+h_i-h_q-1} + \sum_{j=1}^{N_2} B_j u_{t+h_i-L_j})^T M_{t+h_i} B u_{t+h_i} + 0.5u^T_{t+h_i}B^T M_{t+h_i} B u_{t+h_i}$$
$$+ 0.5P_{t+h_i}Bu_{t+h_i} + 0.5P_{t+h_i}(Ax_{t+h_i-1} + \sum_{q=1}^{N_1} A_q x_{t+h_i-h_q-1} + \sum_{j=1}^{N_2} B_j u_{t+h_i-L_j})$$
$$+ 0.5\omega_{t+h_i} + 0.5tr(M_{t+h_i}Q)]\delta(t+h_i)\mathrm{d}u_{t+h_i}\Big]$$
$$= \sum_{i=1}^{N_1} [0.5\bar{\mu}^T_{t+h_i}M_{t+h_i}\bar{\mu}_{t+h_i} + 0.5\omega_{t+h_i} + 0.5tr(M_{t+h_i}Q) + 0.5P_{t+h_i}\bar{\mu}_{t+h_i} + 0.5tr(M_{t+h_i}\Sigma_{t+h_i})]\delta(t+h_i), \tag{36}$$

where we used Equation (17). Similarly, using $\beta_4(.)$ as obtained from (35) in the last equation in (10), yields $\tilde{\beta}_4(.)$,

$$\tilde{\beta}_4(.) = \int c(u_{t+L_j}|x_{t+L_j-1}, x_{t+L_j-h_1-1}, \dots, x_{t+L_j-h_{N_1}-1}, u_{t+L_j-L_1}, \dots, u_{t+L_j-L_{N_2}})\beta_4(.)\delta(t+L_j)\mathrm{d}u_{t+L_j}$$
$$= \sum_{j=1}^{N_2} \int c(u_{t+L_j}|x_{t+L_j-1}, x_{t+L_j-h_1-1}, \dots, x_{t+L_j-h_{N_1}-1}, u_{t+L_j-L_1}, \dots, u_{t+L_j-L_{N_2}})[0.5(Ax_{t+L_j-1}$$
$$+ \sum_{i=1}^{N_1} A_i x_{t+L_j-h_i-1} + Bu_{t+L_j} + \sum_{d=1}^{N_2} B_d u_{t+L_j-L_d})^T M_{t+L_j}(Ax_{t+L_j-1} + \sum_{i=1}^{N_1} A_i x_{t+L_j-h_i-1} + Bu_{t+L_j}$$
$$+ \sum_{d=1}^{N_2} B_d u_{t+L_j-L_d}) + 0.5P_{t+L_j}(Ax_{t+L_j-1} + \sum_{i=1}^{N_1} A_i x_{t+L_j-h_i-1} + Bu_{t+L_j} + \sum_{d=1}^{N_2} B_d u_{t+L_j-L_d}) + 0.5\omega_{t+L_j}]\delta(t+L_j)\mathrm{d}u_{t+L_j}$$
$$= \sum_{j=1}^{N_2} [0.5\bar{\mu}^T_{t+L_j}M_{t+L_j}\bar{\mu}_{t+L_j} + 0.5P_{t+L_j}\bar{\mu}_{t+L_j} + 0.5\omega_{t+L_j} + 0.5tr(\Sigma_{t+L_j}B^T M_{t+L_j}B)]\delta(t+L_j), \tag{37}$$

where $\bar{\mu}_{t+L_j}$ takes the form as Equation (17).

The performance index (18), can then be verified by substituting β_1, β_2, $\tilde{\beta}_3$ and $\tilde{\beta}_4$ into the first equation in (9). Following this substitution, the integration with respect to u_t can be achieved by completing the square with respect to the control input u_t,

yielding,

$$
\begin{aligned}
\gamma(x_{t-1}) = \exp\Bigg\{ & 0.5\left[(Ax_{t-1}+f_t)^T(M_t+R^{-1})B+0.5P_tB-x_r^TR^{-1}B+\rho_{2,t}^T-u_{r;t}^T\Gamma^{-1}\right]^T \\
& \times\left(B^T(M_t+R^{-1})B+\Gamma^{-1}\right)^{-1}\left[(Ax_{t-1}+f_t)^T(M_t+R^{-1})B+0.5P_tB-x_r^TR^{-1}B+\rho_{2,t}^T-u_{r;t}^T\Gamma^{-1}\right] \\
& -0.5(Ax_{t-1}+f_t)^T(R^{-1}+M_t)(Ax_{t-1}+f_t)-0.5u_{r;t}^T\Gamma^{-1}u_r \\
& +(x_r^TR^{-1}-0.5P_t)(Ax_{t-1}+f_t)-0.5\omega_t-0.5tr(M_tQ)-0.5x_r^TR^{-1}x_r+0.5tr(Q(Q^{-1}-R^{-1})) \\
& -\sum_{i=1}^{N_1}\left[0.5\bar{\mu}_{t+h_i}^TM_{t+h_i}\bar{\mu}_{t+h_i}+0.5\omega_{t+h_i}+0.5tr(M_{t+h_i}Q)+0.5P_{t+h_i}\bar{\mu}_{t+h_i}+0.5tr(M_{t+h_i}\Sigma_{t+h_i})\right]\delta(t+h_i) \\
& -\sum_{j=1}^{N_2}\Bigg[0.5(Ax_{t+L_j-1}+B\bar{u}_{t+L_j}+\sum_{i=1}^{N_1}A_ix_{t+L_j-h_i-1}+\sum_{d=1,L_d\neq L_j}^{N_2}B_du_{t+L_j-L_d})^TM_{t+L_j}\bar{\mu}_{t+L_j} \\
& +0.5P_{t+L_j}(Ax_{t+L_j-1}+B\bar{u}_{t+L_j}+\sum_{i=1}^{N_1}A_ix_{t+L_j-h_i-1}+\sum_{d=1,L_d\neq L_j}^{N_2}B_du_{t+L_j-L_d})+0.5\omega_{t+L_j}+0.5tr(\Sigma_{t+L_j}B^TM_{t+L_j}B)\Bigg]\delta(t+L_j)\Bigg\},
\end{aligned}
\tag{38}
$$

where the item,

$$
-\sum_{i=1}^{N_1}\left[0.5\bar{\mu}_{t+h_i}^TM_{t+h_i}\bar{\mu}_{t+h_i}+0.5\omega_{t+h_i}+0.5tr(M_{t+h_i}Q)+0.5P_{t+h_i}\bar{\mu}_{t+h_i}+0.5tr(M_{t+h_i}\Sigma_{t+h_i})\right]\delta(t+h_i),
$$

in Equation (38) can be further extended as follows,

$$
\begin{aligned}
& -\sum_{i=1}^{N_1}\left[0.5\bar{\mu}_{t+h_i}^TM_{t+h_i}\bar{\mu}_{t+h_i}+0.5\omega_{t+h_i}+0.5tr(M_{t+h_i}Q)+0.5P_{t+h_i}\bar{\mu}_{t+h_i}+0.5tr(M_{t+h_i}\Sigma_{t+h_i})\right]\delta(t+h_i) \\
& =-\sum_{i=1}^{N_1}\Bigg[0.5\bar{\mu}_{t+h_i}^TM_{t+h_i}A_ix_{t-1}+0.5\bar{\mu}_{t+h_i}^TM_{t+h_i}(Ax_{t+h_i-1}+\sum_{q=1,h_i\neq h_q}^{N_1}A_qx_{t+h_i-h_q-1} \\
& +B\bar{u}_{t+h_i}+\sum_{j=1}^{N_2}B_ju_{t+h_i-L_j})+0.5\omega_{t+h_i}+0.5tr(M_{t+h_i}Q)++0.5tr(M_{t+h_i}\Sigma_{t+h_i})+0.5P_{t+h_i}A_ix_{t-1} \\
& +0.5P_{t+h_i}(Ax_{t+h_i-1}+\sum_{q=1,h_i\neq h_q}^{N_1}A_qx_{t+h_i-h_q-1}+B\bar{u}_{t+h_i}+\sum_{j=1}^{N_2}B_ju_{t+h_i-L_j})\Bigg]\delta(t+h_i).
\end{aligned}
\tag{39}
$$

By substituting Equation (39) back into Equation (38), the final form of $\gamma(x_{t-1})$ can be specified as follows,

$$
\begin{aligned}
\gamma(x_{t-1}) &= \exp\Bigg\{-0.5x_{t-1}^T A^T\Bigg[-(M_t+R^{-1})B\Big(B^T(M_t+R^{-1})B+\Gamma^{-1}\Big)^{-1}B^T(M_t+R^{-1}) \\
&+(R^{-1}+M_t)\Bigg]Ax_{t-1}-\Bigg[-\Big(f_t^T(M_t+R^{-1})B+0.5P_tB-x_r^TR^{-1}B+\rho_{2,t}^T-u_{r;t}^T\Gamma^{-1}\Big)\Big(B^T(M_t+R^{-1})B+\Gamma^{-1}\Big)^{-1} \\
&\times B^T(M_t+R^{-1})A+f_t^T(R^{-1}+M_t)A+\rho_{1,t}-(x_r^TR^{-1}-0.5P_t)A\Bigg]x_{t-1} \\
&+0.5\Big[f_t^T(M_t+R^{-1})B+0.5P_tB-x_r^TR^{-1}B+\rho_{2,t}^T-u_{r;t}^T\Gamma^{-1}\Big]^T\Big(B^T(M_t+R^{-1})B+\Gamma^{-1}\Big)^{-1} \\
&\times\Big[f_t^T(M_t+R^{-1})B+0.5P_tB-x_r^TR^{-1}B+\rho_{2,t}^T-u_{r;t}^T\Gamma^{-1}\Big]-0.5f_t^T(R^{-1}+M_t)f_t^T-0.5u_{r;t}^T\Gamma^{-1}u_r \\
&+(x_r^TR^{-1}-0.5P_t)f_t-0.5\omega_t-0.5tr(M_tQ)-0.5x_r^TR^{-1}x_r+0.5tr(Q(Q^{-1}-R^{-1})) \\
&-\sum_{i=1}^{N_1}\Bigg[0.5\bar{\mu}_{t+h_i}^TM_{t+h_i}(Ax_{t+h_i-1}+\sum_{q=1,h_i\neq h_q}^{N_1}A_qx_{t+h_i-h_q-1}+B\bar{u}_{t+h_i}+\sum_{j=1}^{N_2}B_ju_{t+h_i-L_j})+0.5\omega_{t+h_i}+0.5tr(M_{t+h_i}Q) \\
&+0.5tr(M_{t+h_i}\Sigma_{t+h_i})+0.5P_{t+h_i}(Ax_{t+h_i-1}+\sum_{q=1,h_i\neq h_q}^{N_1}A_qx_{t+h_i-h_q-1}+B\bar{u}_{t+h_i}+\sum_{j=1}^{N_2}B_ju_{t+h_i-L_j})\Bigg]\delta(t+h_i) \\
&-\sum_{j=1}^{N_2}\Bigg[0.5(Ax_{t+L_j-1}+B\bar{u}_{t+L_j}+\sum_{i=1}^{N_1}A_ix_{t+L_j-h_i-1}+\sum_{d=1,L_d\neq L_j}^{N_2}B_du_{t+L_j-L_d})^TM_{t+L_j}\bar{\mu}_{t+L_j} \\
&+0.5P_{t+L_j}(Ax_{t+L_j-1}+B\bar{u}_{t+L_j}+\sum_{i=1}^{N_1}A_ix_{t+L_j-h_i-1}+\sum_{d=1,L_d\neq L_j}^{N_2}B_du_{t+L_j-L_d})+0.5\omega_{t+L_j}+0.5tr(\Sigma_{t+L_j}B^TM_{t+L_j}B)\Bigg]\delta(t+L_j)\Bigg\}
\end{aligned}
\tag{40}
$$

Equating quadratic and linear terms in x_{t-1} with the quadratic and linear terms in x_{t-1} of Equation (18) yields the forms of M_{t-1} and P_{t-1} as specified by Equations (19) and (20) respectively. Similarly the constant terms which are independent of the state x_{t-1} can be equated to ω_{t-1} yielding the form of ω_{t-1} as specified by Equation (21).

To obtain the distribution of the optimal controller, the parameters β_1, β_2, $\tilde{\beta}_3$, $\tilde{\beta}_4$ and $\gamma(x_{t-1})$ are substituted into Equation (8), to give,

$$
\begin{aligned}
&c(u_t \mid x_{t-1},x_{t-h_1},\ldots,x_{t-h_{N_1}},u_{t-L_1},\ldots,u_{t-L_{N_2}}) \\
&=\frac{{}^Ic(u_t|x_{t-1},x_{t-h_1},\ldots,x_{t-h_{N_1}},u_{t-L_1},\ldots,u_{t-L_{N_2}})\exp\Big[-\beta_1(.)-\beta_2(.)-\tilde{\beta}_3(.)-\tilde{\beta}_4(.)\Big]}{\gamma(x_{t-1})} \\
&=\exp\Bigg\{-\Bigg[u_t+\Big(B^T(M_t+R^{-1})B+\Gamma^{-1}\Big)^{-1}\Big[(Ax_{t-1}+f_t)^T(M_t+R^{-1})B+0.5P_tB \\
&-x_r^TR^{-1}B+\rho_{2,t}^T-u_{r;t}^T\Gamma^{-1}\Big]\Bigg]^T\Big(M_t+R^{-1})B+\Gamma^{-1}\Big)\Bigg[u_t+\Big(B^T(M_t+R^{-1})B+\Gamma^{-1}\Big)^{-1} \\
&\times\Big[(Ax_{t-1}+f_t)^T(M_t+R^{-1})B+0.5P_tB-x_r^TR^{-1}B+\rho_{2,t}^T-u_{r;t}^T\Gamma^{-1}\Big]\Bigg]\Bigg\},
\end{aligned}
\tag{41}
$$

which is a Gaussian distribution with mean and covariance matrix as specified by (17). End of proof.
